# Deep learning with convolutional neural networks for EEG decoding and visualization

**DOI:** 10.1002/hbm.23730

**Published:** 2017-08-07

**Authors:** Robin Tibor Schirrmeister, Jost Tobias Springenberg, Lukas Dominique Josef Fiederer, Martin Glasstetter, Katharina Eggensperger, Michael Tangermann, Frank Hutter, Wolfram Burgard, Tonio Ball

**Affiliations:** ^1^ Translational Neurotechnology Lab, Epilepsy Center, Medical Center – University of Freiburg, Engelberger Str. 21 Freiburg 79106 Germany; ^2^ BrainLinks‐BrainTools Cluster of Excellence, University of Freiburg, Georges‐Köhler‐Allee 79 Freiburg 79110 Germany; ^3^ Machine Learning Lab Computer Science Dept, University of Freiburg, Georges‐Köhler‐Allee 79 Freiburg 79110 Germany; ^4^ Neurobiology and Biophysics Faculty of Biology, University of Freiburg, Hansastr. 9a Freiburg 79104 Germany; ^5^ Machine Learning for Automated Algorithm Design Lab Computer Science Dept, University of Freiburg, Georges‐Köhler‐Allee 52 Freiburg im Breisgau 79110 Germany; ^6^ Brain State Decoding Lab Computer Science Dept, University of Freiburg, Albertstr. 23 Freiburg 79104 Germany; ^7^ Autonomous Intelligent Systems Lab Computer Science Dept, University of Freiburg, Georges‐Köhler‐Allee 79 Freiburg 79110 Germany

**Keywords:** electroencephalography, EEG analysis, machine learning, end‐to‐end learning, brain–machine interface, brain–computer interface, model interpretability, brain mapping

## Abstract

Deep learning with convolutional neural networks (deep ConvNets) has revolutionized computer vision through end‐to‐end learning, that is, learning from the raw data. There is increasing interest in using deep ConvNets for end‐to‐end EEG analysis, but a better understanding of how to design and train ConvNets for end‐to‐end EEG decoding and how to visualize the informative EEG features the ConvNets learn is still needed. Here, we studied deep ConvNets with a range of different architectures, designed for decoding imagined or executed tasks from raw EEG. Our results show that recent advances from the machine learning field, including batch normalization and exponential linear units, together with a cropped training strategy, boosted the deep ConvNets decoding performance, reaching at least as good performance as the widely used filter bank common spatial patterns (FBCSP) algorithm (mean decoding accuracies 82.1% FBCSP, 84.0% deep ConvNets). While FBCSP is designed to use spectral power modulations, the features used by ConvNets are not fixed a priori. Our novel methods for visualizing the learned features demonstrated that ConvNets indeed learned to use spectral power modulations in the alpha, beta, and high gamma frequencies, and proved useful for spatially mapping the learned features by revealing the topography of the causal contributions of features in different frequency bands to the decoding decision. Our study thus shows how to design and train ConvNets to decode task‐related information from the raw EEG without handcrafted features and highlights the potential of deep ConvNets combined with advanced visualization techniques for EEG‐based brain mapping. *Hum Brain Mapp 38:5391–5420, 2017*. © **2017 Wiley Periodicals, Inc.**

## INTRODUCTION

Machine‐learning techniques allow extracting information from electroencephalographic (EEG) recordings of brain activity, and therefore play a crucial role in several important EEG‐based research and application areas. For example, machine‐learning techniques are a central component of many EEG‐based brain‐computer interface (BCI) systems for clinical applications. Such systems already allowed, for example, persons with severe paralysis to communicate [Nijboer et al., 2008], to draw pictures [Münßinger et al., 2010], and to control telepresence robots [Tonin et al., 2011]. Such systems may also facilitate stroke rehabilitation [Ramos‐Murguialday et al., 2013] and may be used in the treatment of epilepsy [Gadhoumi et al., 2016] (for more examples of potential clinical applications, see Moghimi et al. [2013]). Furthermore, machine‐learning techniques for the analysis of brain signals, including the EEG, are increasingly recognized as novel tools for neuroscientific inquiry [Das et al., 2010; Knops et al., 2009; Kurth‐Nelson et al., 2016; Stansbury et al., 2013].

However, despite many examples of impressive progress, there is still room for considerable improvement with respect to several important aspects of information extraction from the EEG, including its accuracy, interpretability, and usability for online applications. Therefore, there is a continued interest in transferring innovations from the area of machine learning to the fields of EEG decoding and BCI. A recent, prominent example of such an advance in machine learning is the application of convolutional neural networks (ConvNets), particularly in computer vision tasks. Thus, first studies have started to investigate the potential of ConvNets for brain‐signal decoding [Antoniades et al., 2016; Bashivan et al., 2016; Cecotti and Graser, 2011; Hajinoroozi et al., 2016; Lawhern et al., 2016; Liang et al., 2016; Manor et al., 2016; Manor and Geva, 2015; Page et al., 2016; Ren and Wu, 2014; Sakhavi et al., 2015; Shamwell et al., 2016; Stober, 2016; Stober et al., 2014; Sun et al., 2016; Tabar and Halici, 2017; Tang et al., 2017; Thodoroff et al., 2016; Wang et al., 2013] (see Supporting Information, Section A.1 for more details on these studies). Still, several important methodological questions on EEG analysis with ConvNets remain, as detailed below and addressed in this study.

ConvNets are artificial neural networks that can learn local patterns in data by using convolutions as their key component (also see the section “Convolutional Neural Networks”). ConvNets vary in the number of convolutional layers, ranging from shallow architectures with just one convolutional layer such as in a successful speech recognition ConvNet [Abdel‐Hamid et al., 2014] over deep ConvNets with multiple consecutive convolutional layers [Krizhevsky et al., 2012] to very deep architectures with more than 1000 layers as in the case of the recently developed residual networks [He et al., 2015]. Deep ConvNets can first extract local, low‐level features from the raw input and then increasingly more global and high level features in deeper layers. For example, deep ConvNets can learn to detect increasingly complex visual features (e.g., edges, simple shapes, complete objects) from raw images. Over the past years, deep ConvNets have become highly successful in many application areas, such as in computer vision and speech recognition, often outperforming previous state‐of‐the‐art methods (we refer to LeCun et al. [2015] and Schmidhuber [2015] for recent reviews). For example, deep ConvNets reduced the error rates on the ImageNet image‐recognition challenge, where 1.2 million images must be classified into 1000 different classes, from above 26% to below 4% within 4 years [He et al., 2015; Krizhevsky et al., 2012]. ConvNets also reduced error rates in recognizing speech, for example, from English news broadcasts [Sainath et al., 2015a,2015c; Sercu et al., 2016]; however, in this field, hybrid models combining ConvNets with other machine‐learning components, notably recurrent networks, and deep neural networks without convolutions are also competitive [Li and Wu, 2015; Sainath et al., 2015b; Sak et al., 2015]. Deep ConvNets also contributed to the spectacular success of AlphaGo, an artificial intelligence that beat the world champion in the game of Go [Silver et al., 2016].

ConvNets have both advantages and disadvantages compared to other machine learning models. Advantages of ConvNets include that they are well suited for end‐to‐end learning, that is, learning from the raw data without any *a priori* feature selection, that they scale well to large datasets, and that they can exploit hierarchical structure in natural signals. Disadvantages of ConvNets include that they may output false predictions with high confidence [Nguyen et al., 2015; Szegedy et al., 2014] may require a large amount of training data, may take longer to train than simpler models, and involve a large number of hyperparameters such as the number of layers or the type of activation functions. Deep ConvNets are also notoriously difficult to interpret. In the light of these advantages and disadvantages, in this study, we focused on how ConvNets of different architectures can be designed and trained for end‐to‐end learning of EEG recorded in human subjects, and how they can be made more interpretable via suitable visualization techniques.

The EEG signal has characteristics that make it different from inputs that ConvNets have been most successful on, namely images. In contrast to two‐dimensional static images, the EEG signal is a dynamic time series from electrode measurements obtained on the three‐dimensional scalp surface. Also, the EEG signal has a comparatively low signal‐to‐noise ratio, that is, sources that have no task‐relevant information often affect the EEG signal more strongly than the task‐relevant sources. These properties could make learning features in an end‐to‐end fashion fundamentally more difficult for EEG signals than for images. Thus, the existing ConvNets architectures from the field of computer vision need to be adapted for EEG input and the resulting decoding accuracies rigorously evaluated against more traditional feature extraction methods. For that purpose, a well‐defined baseline is crucial, that is, a comparison against an implementation of a standard EEG decoding method validated on published results for that method. In light of this, in this study, we addressed two key questions:

- What is the impact of ConvNet *design choices* (e.g., the overall network architecture or other design choices such as the type of nonlinearity used) on the decoding accuracies?
- What is the impact of ConvNet *training strategies* (e.g., training on entire trials or crops within trials) on the decoding accuracies?

To address these questions, we created three ConvNets with different architectures, with the number of convolutional layers ranging from 2 layers in a “shallow” ConvNet over a 5‐layer deep ConvNet up to a 31‐layer residual network (ResNet). Additionally, we also created a hybrid ConvNet from the deep and shallow ConvNets. As described in detail in the methods section, these architectures were inspired both from existing “non‐ConvNet” EEG decoding methods, which we embedded in a ConvNet, and from previously published successful ConvNet solutions in the image processing domain (e.g., the ResNet architecture recently won several image recognition competitions [He et al., 2015]). All architectures were adapted to the specific requirements imposed by the analysis of multi‐channel EEG data. To address whether these ConvNets can reach competitive decoding accuracies, we performed a statistical comparison of their decoding accuracies to those achieved with decoding based on filter bank common spatial patterns (FBCSP) [Ang et al., 2008; Chin et al., 2009], a method that is widely used in EEG decoding and has won several EEG decoding competitions such as BCI competition IV datasets 2a and 2b. We analyzed the offline decoding performance on four suitable EEG decoding datasets (see the section “Datasets and Preprocessing” for details). In all cases, we used only minimal preprocessing to conduct a fair end‐to‐end comparison of ConvNets and FBCSP.

In addition to the role of the overall network architecture, we systematically evaluated a range of important design choices. We focused on alternatives resulting from recent advances in machine‐learning research on deep ConvNets. Thus, we evaluated potential performance improvements by using dropout as a novel regularization strategy [Srivastava et al., 2014], intermediate normalization by batch normalization [Ioffe and Szegedy, 2015] or exponential linear units as a recently proposed activation function [Clevert et al., 2016]. A comparable analysis of the role of deep ConvNet design choices in EEG decoding is currently lacking.

In addition to the global architecture and specific design choices which together define the “structure” of ConvNets, another important topic that we address is how a given ConvNet should be trained on the data. As with architecture and design, there are several different methodological options and choices with respect to the training process, such as the optimization algorithm (e.g., Adam [Kingma and Ba, 2014], Adagrad [Duchi et al., 2011], etc.), or the sampling of the training data. Here, we focused on the latter question of sampling the training data as there is usually, compared to current computer vision tasks with millions of samples, relatively little data available for EEG decoding. Therefore, we evaluated two sampling strategies, both for the deep and shallow ConvNets: training on whole trials or on multiple crops of the trial, that is, on windows shifted through the trials. Using multiple crops holds promise as it increases the amount of training examples, which has been crucial to the success of deep ConvNets. Using multiple crops has become standard procedure for ConvNets for image recognition [He et al., 2015; Howard, 2013; Szegedy et al., 2015], but the usefulness of cropped training has not yet been examined in EEG decoding.

In addition to the problem of achieving good decoding accuracies, a growing corpus of research tackles the problem of understanding what ConvNets learn (see Yeager [2016] for a recent overview). This direction of research may be especially relevant for neuroscientists interested in using ConvNets—insofar as they want to understand what features in the brain signal discriminate the investigated classes. Here we present two novel methods for *feature visualization* that we used to gain insights into our ConvNet learned from the neuronal data.

We concentrated on EEG band power features as a target for visualizations. Based on a large body of literature on movement‐related spectral power modulations [Chatrian et al., 1959; Pfurtscheller and Aranibar, 1977, 1978; Pfurtscheller and Berghold, 1989; Pfurtscheller et al., 1994; Toro et al., 1994], we had clear expectations which band power features should be discriminative for the different classes. The motivation for developing our visualization methods was threefold:

1. Verify that the ConvNets are using actual brain signals
2. Gain insights into the ConvNet behavior, e.g., what EEG features the ConvNet uses to decode the signal
3. Potentially make steps toward using ConvNets for brain mapping.

Our first method can be used to show how much information about a specific feature is retained in the ConvNet in different layers; however, it does not evaluate whether the feature causally affects the ConvNet outputs. Therefore, we designed our second method to directly investigate causal effects of the feature values on the ConvNet outputs. With both visualization methods, it is possible to derive topographic scalp maps that either show how much information about the band power in different frequency bands is retained in the outputs of the trained ConvNet or how much they causally affect the outputs of the trained ConvNet.

Addressing the questions raised above, in summary the main contributions of this study are as follows:

- We show for the first time that within‐subject end‐to‐end‐trained deep ConvNets can reach accuracies at least in the same range as FBCSP for decoding task‐related information from EEG.
- We evaluate a large number of ConvNet design choices on an EEG decoding task, and we show that recently developed methods from the field of deep learning such as batch normalization and exponential linear units are crucial for reaching high decoding accuracies.
- We show that cropped training can increase the decoding accuracy of deep ConvNets and describe a computationally efficient training strategy to train ConvNets on a larger number of input crops per EEG trial.
- We develop and apply novel visualizations that highly suggest that the deep ConvNets learn to use the band power in frequency bands relevant for motor decoding (alpha, beta, and gamma) with meaningful spatial distributions.

Thus, in summary, the methods and findings described in this study are a first step and preliminary approximation to a comprehensive investigation of the role of deep ConvNet design choices, training strategies and visualization techniques for EEG decoding and pave the way for a more widespread application both in clinical applications and neuroscientific research.

## METHODS

We first provide basic definitions with respect to brain‐signal decoding as a supervised classification problem used in the remaining Methods section. This is followed by the principles of both filter bank common spatial patterns (FBCSP), the established baseline decoding method referred to throughout this study, and of convolutional neural networks (ConvNets). Next, we describe the ConvNets developed for this study in detail, including the *design choices* we evaluated. Afterward, the training of the ConvNets, including two *training strategies*, is described. Then we present two novel *visualizations of trained ConvNets* in the section “Visualization”. Datasets, preprocessing descriptions and statistical evaluation methods follow in the sections “Datasets and Preprocessing” and “Statistics.” Details about software and hardware can be found in Supporting Information, Sections 2.8 and A.8. The code used in this study is available under https://github.com/robintibor/braindecode/.

### Definitions and Notation

This section more formally defines how brain‐signal decoding can be viewed as a supervised classification problem and includes the notation used to describe the methods.

#### Input and labels

We assume that we are given one EEG dataset per subject *i*. Each dataset is separated into labeled trials (time‐segments of the original recording that each belong to one of several classes). Concretely, we are given datasets $D^{i}=\{(X^{1},y^{1}),\ldots ,(X^{N_{i}},y^{N_{i}})\}$, where *N_i_* denotes the total number of recorded trials for subject *i*. The input matrix $X^{j}\in {\mathbb{R}}^{E\cdot T}$ of trial $j,1\leq j\leq N_{i}$ contains the preprocessed signals of *E*‐recorded electrodes and *T*‐discretized time steps recorded per trial.

The corresponding class label of trial *j* is denoted by *y^j^*. It takes values from a set of *K* class labels *L* that, in our case, correspond to the imagined or executed movements performed in each trial, for example, $\forall y^{j}:y^{j}\in L=\{l_{1}=“``\text{Hand}\;(\text{Left})”,l_{2}=``“\text{Hand}\;(\text{Right})”,l_{3}=“``\text{Feet}”,l_{4}=``“\text{Rest}”\}$.

#### Decoder

The decoder *f* is trained on these existing trials such that it is able to assign the correct label to new unseen trials. Concretely, we aim to train the decoder to assign the label *y^j^* to trial *X^j^* using the output of a parametric classifier $f(X^{j};\theta ):{\mathbb{R}}^{E\cdot T}\to L$ with parameters *θ*.

For the rest of this article, we assume that the classifier $f(X^{j};\theta )$ is represented by a standard machine‐learning pipeline decomposed into two parts: a first part that extracts a (vector‐valued) feature representation $\phi (X^{j};\theta_{\phi })$ with parameters $\theta_{\phi }$—which could either be set manually (for hand designed features), or learned from the data; and a second part consisting of a classifier g with parameters *θ_g_* that is trained using these features, that is, $f(X^{j};\theta )=g(\phi (X^{j};\theta_{\phi }),\theta_{g})$.

As described in detail in the following sections, it is important to note that FBCSP and ConvNets differ in how they implement this framework: in short, FBCSP has separated feature extraction and classifier stages, while ConvNets learn both stages jointly.

### Filter Bank Common Spatial Patterns (FBCSP)

FBCSP [Ang et al., 2008; Chin et al., 2009] is a widely used method to decode oscillatory EEG data, for example, with respect to movement‐related information, that is, the decoding problem we focus on in this study. FBCSP was the best‐performing method for the BCI competition IV dataset 2a, which we use in this study (see the section “Datasets and Preprocessing” for a short dataset description). FBCSP also won other similar EEG decoding competitions [Tangermann et al., 2012]. Therefore, we consider FBCSP an adequate benchmark algorithm for the evaluation of the performance of ConvNets in this study.

In the following, we explain the computational steps of FBCSP. We will refer to these steps when explaining our shallow ConvNet architecture (see the section “Shallow ConvNet for raw EEG signals”), as it is inspired by these steps.

In a supervised manner, FBCSP computes spatial filters (linear combinations of EEG channels) that enhance class‐discriminative band power features contained in the EEG. FBCSP extracts and uses these features $\phi (X^{j};\theta_{\phi })$ (which correspond to the feature representation part in the section “Decoder”) to decode the label of a trial in several steps (we will refer back to these steps when we explain the shallow ConvNet):

1. **Bandpass filtering**: Different bandpass filters are applied to separate the raw EEG signal into different frequency bands.
2. **Epoching**: The continuous EEG signal is cut into trials as explained in the section “Input and labels.”
3. **CSP computation**: Per frequency band, the common spatial patterns (CSP) algorithm is applied to extract spatial filters. CSP aims to extract spatial filters that make the trials discriminable by the power of the spatially filtered trial signal (see Koles et al. [1990], Ramoser et al. [2000], and Blankertz et al. [2008] for more details on the computation). The spatial filters correspond to the learned parameters $\theta_{\phi }$ in FBCSP.
4. **Spatial filtering**: The spatial filters computed in Step 2 are applied to the EEG signal.
5. **Feature construction**: Feature vectors $\phi (X^{j};\theta_{\phi })$ are constructed from the filtered signals: Specifically, feature vectors are the log‐variance of the spatially filtered trial signal for each frequency band and for each spatial filter.
6. **Classification**: A classifier is trained to predict per‐trial labels based on the feature vectors.

For details on our FBCSP implementation, see Supporting Information, Section A.2.

### Convolutional Neural Networks

In the following sections, we first explain the basic ideas of ConvNets. We then describe architectural choices for ConvNets on EEG, including how to represent the EEG input for a ConvNet, the three different ConvNet architectures used in this study and several specific design choices that we evaluated for these architectures. Finally, we describe how to train the ConvNets, including the description of a trial‐wise and a cropped training strategy for our EEG data.

#### Basics

Generally, ConvNets combine two ideas useful for many learning tasks on natural signals, such as images and audio signals. These signals often have an inherent hierarchical structure (e.g., images typically consist of edges that together form simple shapes which again form larger, more complex shapes and so on). ConvNets can learn local non‐linear features (through convolutions and nonlinearities) and represent higher‐level features as compositions of lower level features (through multiple layers of processing). In addition, many ConvNets use pooling layers which create a coarser intermediate feature representation and can make the ConvNet more translation invariant. For further details, see LeCun et al. [2015], Goodfellow et al. [2016], and Schmidhuber [2015].

### ConvNet Architectures and Design Choices

#### Input representation

The first important decision for applying ConvNets to EEG decoding is how to represent the input $X^{j}\in {\mathbb{R}}^{E\cdot T}$. One possibility would be to represent the EEG as a time series of topographically organized images, that is, of the voltage distributions across the (flattened) scalp surface (this has been done for ConvNets that get power spectra as input [Bashivan et al., 2016]). However, EEG signals are assumed to approximate a linear superposition of spatially global voltage patterns caused by multiple dipolar current sources in the brain [Nunez and Srinivasan, 2006]. Unmixing of these global patterns using a number of spatial filters is therefore typically applied to the whole set of relevant electrodes as a basic step in many successful examples of EEG decoding [Ang et al., 2008; Blankertz et al., 2008; Rivet et al., 2009].

In this view, all relevant EEG modulations are global in nature, due to the physical origin of the noninvasive EEG and hence there would be no obvious hierarchical compositionality of local and global EEG modulations *in space*. In contrast, there is an abundance of evidence that the EEG is organized across multiple time scales, such as in nested oscillations [Canolty et al., 2006; Monto et al., 2008; Schack et al., 2002; Vanhatalo et al., 2004] involving both local and global modulations *in time*. In light of this, we designed ConvNets in a way that they can learn spatially global unmixing filters in the entrance layers, and temporal hierarchies of local and global modulations in the deeper architectures. To this end, we represent the input as a 2D‐array with the number of time steps as the width and the number of electrodes as the height. This approach also significantly reduced the input dimensionality compared with the “EEG‐as‐an‐image” approach.

#### Deep ConvNet for raw EEG signals

To tackle the task of EEG decoding, we designed a deep ConvNet architecture inspired by successful architectures in computer vision, as for example described in Krizhevsky et al. [2012]. The requirements for this architecture are as follows: We want a model that is able to extract a wide range of features and is not restricted to specific feature types [Hertel et al., 2015]. We were interested in such a generic architecture for two reasons: (1) we aimed to uncover whether such a generic ConvNet designed only with minor expert knowledge can reach competitive accuracies, and, (2) to lend support to the idea that standard ConvNets can be used as a general‐purpose tool for brain‐signal decoding tasks. As an aside, keeping the architecture generic also increases the chances that ConvNets for brain decoding can directly profit from future methodological advances in deep learning.

**Figure 1:**
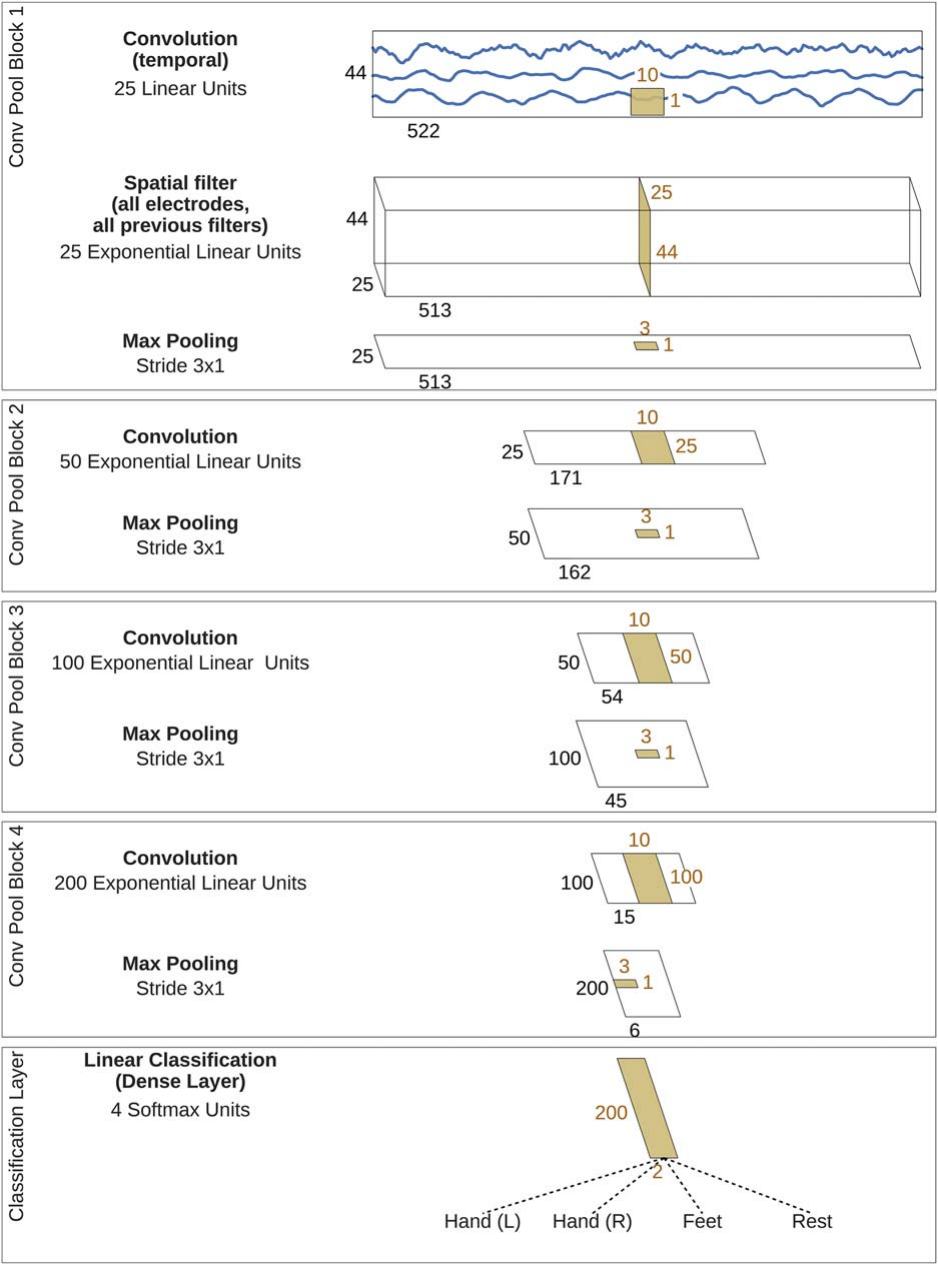
Deep ConvNet architecture. EEG input (at the top) is progressively transformed toward the bottom, until the final classifier output. Black cuboids: inputs/feature maps; brown cuboids: convolution/pooling kernels. The corresponding sizes are indicated in black and brown, respectively. Sizes are for the cropped training version, see the section “Architecture differences.” Each spatial filter has weights for all possible pairs of electrodes with filters of the preceding temporal convolution. Note that in these schematics, proportions of maps and kernels are only approximate. [Color figure can be viewed at http://wileyonlinelibrary.com]

Our deep ConvNet had four convolution‐max‐pooling blocks, with a special first block designed to handle EEG input (see below), followed by three standard convolution‐max‐pooling blocks and a dense softmax classification layer (Fig. 1).

The first convolutional block was split into two layers in order to better handle the large number of input channels—one input channel per electrode compared to three input channels (one per color) in rgb‐images. In the first layer, each filter performs a convolution over time, and in the second layer, each filter performs a spatial filtering with weights for all possible pairs of electrodes with filters of the preceding temporal convolution. Note that as there is no activation function in between the two layers, they could in principle be combined into one layer. Using two layers however implicitly regularizes the overall convolution by forcing a separation of the linear transformation into a combination of a temporal convolution and a spatial filter. This splitted convolution was evaluated against a single‐step convolution in our experiments (see the section “Design choices for deep and shallow ConvNet”).

We used exponential linear units (ELUs, *f*(*x*) = *x* for *x* > 0 and $f(x)=e^{x}-1$ for x<=0 [Clevert et al., 2016]) as activation functions (we also evaluated Rectified Linear Units (ReLUs, f(x)=max(x,0)), as a less recently proposed alternative, see the section “Design choices for deep and shallow ConvNet”).

#### Shallow ConvNet for raw EEG signals

**Figure 2:**
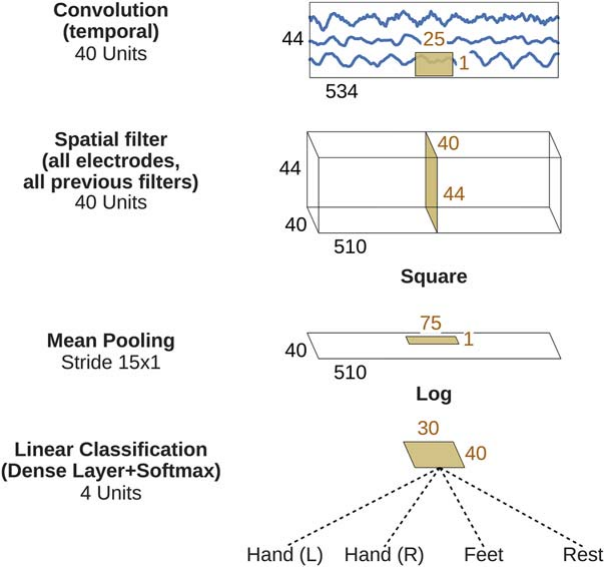
Shallow ConvNet architecture. Conventions as in Figure 1. [Color figure can be viewed at http://wileyonlinelibrary.com]

We also designed a more shallow architecture referred to as shallow ConvNet, inspired by the FBCSP pipeline (Fig. 2), specifically tailored to decode band power features. The transformations performed by the shallow ConvNet are similar to the transformations of FBCSP (see the section “Filter Bank Common Spatial Patterns (FBCSP)”). Concretely, the first two layers of the shallow ConvNet perform a temporal convolution and a spatial filter, as in the deep ConvNet. These steps are analogous to the bandpass and CSP spatial filter steps in FBCSP. In contrast to the deep ConvNet, the temporal convolution of the shallow ConvNet had a larger kernel size (25 vs 10), allowing a larger range of transformations in this layer (smaller kernel sizes for the shallow ConvNet led to lower accuracies in preliminary experiments on the training set). After the temporal convolution and the spatial filter of the shallow ConvNet, a squaring nonlinearity, a mean pooling layer and a logarithmic activation function followed; together these steps are analogous to the trial log‐variance computation in FBCSP (we note that these steps were not used in the deep ConvNet). In contrast to FBCSP, the shallow ConvNet embeds all the computational steps in a single network, and thus all steps can be optimized jointly (see the section “ConvNet Training”). Also, due to having several pooling regions within one trial, the shallow ConvNet can learn a temporal structure of the band power changes within the trial, which was shown to help classification in prior work [Sakhavi et al., 2015].

#### Design choices for deep and shallow ConvNet

For both architectures described above, we evaluated several design choices. We evaluated architectural choices which we expect to have a potentially large impact on the decoding accuracies and/or from which we hoped to gain insights into the behavior of the ConvNets. Thus, for the deep ConvNet, we compared the design aspects listed in Table 1.

**Table 1:** Evaluated design choices

Design aspect	Our choice	Variants	Motivation
Activation functions Pooling mode	ELU Max	Square, ReLU Mean	We expected these choices to be sensitive to the type of feature (e.g., signal phase or power), as squaring and mean pooling results in mean power (given a zero‐mean signal). Different features may play different roles in the low‐frequency components vs the higher frequencies (see the section “Datasets and Preprocessing”).
Regularization and intermediate normalization	Dropout + batch normalization + a new tied loss function (explanations see text)	Only batch normalization, only dropout, neither of both, nor tied loss	We wanted to investigate whether recent deep learning advances improve accuracies and check how much regularization is required.
Factorized temporal convolutions	One 10 × 1 convolution per convolutional layer	Two 6 × 1 convolutions per convolutional layer	Factorized convolutions are used by other successful ConvNets [Szegedy et al., 2015]
Splitted vs one‐step convolution	Splitted convolution in first layer (see the section “Deep ConvNet for raw EEG signals”)	One‐step convolution in first layer	Factorizing convolution into spatial and temporal parts may improve accuracies for the large number of EEG input channels (compared with three rgb color channels of regular image datasets).

Design choices we evaluated for our convolutional networks. “Our choice” is the choice we used when evaluating ConvNets in the remainder of this article, for example, versus FBCSP. Note that these design choices have not been evaluated in prior work, see Supporting Information, Section A.1.

In the following, we give additional details for some of these aspects. Batch normalization standardizes intermediate outputs of the network to zero mean and unit variance for a batch of training examples [Ioffe and Szegedy, 2015]. This is meant to facilitate the optimization by keeping the inputs of layers closer to a normal distribution during training. We applied batch normalization, as recommended in the original paper [Ioffe and Szegedy, 2015], to the output of convolutional layers before the nonlinearity. Dropout randomly sets some inputs for a layer to zero in each training update. It is meant to prevent co‐adaption of different units and can be seen as analogous to training an ensemble of networks. We drop out the inputs to all convolutional layers after the first with a probability of 0.5. Finally, our new tied loss function is designed to further regularize our cropped training (see the section “Cropped training” for an explanation).

We evaluated the same design aspects for the shallow ConvNet, except for the following differences:

- The baseline methods for the activation function and pooling mode were chosen as “squaring nonlinearity” and “mean pooling,” motivation is given in the section “Shallow ConvNet for raw EEG signals.”
- We did not include factorized temporal convolutions into the comparison, as the longer kernel lengths of the shallow ConvNet make these convolutions less similar to other successful ConvNets anyways.
- We additionally compared the logarithmic nonlinearity after the pooling layer with a square root nonlinearity to check if the logarithmic activation inspired by FBCSP is better than traditional L2‐pooling.

#### Hybrid ConvNet

Besides the individual design choices for the deep and shallow ConvNet, a natural question to ask is whether both ConvNets can be combined into a single ConvNet. Such a hybrid ConvNet could profit from the more specific feature extraction of the shallow ConvNet and from the more generic feature extraction of the deep ConvNet. Therefore, we also created a hybrid ConvNet by fusing both networks after the final layer. Concretely, we replaced the four‐filter softmax classification layers of both ConvNets by 60‐ and 40‐filter ELU layers for the deep and shallow ConvNet respectively. The resulting 100 feature maps were concatenated and used as the input to a new softmax classification layer. We retrained the whole hybrid ConvNet from scratch and did not use any pretrained deep or shallow ConvNet parameters.

#### Residual ConvNet for raw EEG signals

**Figure 3:**
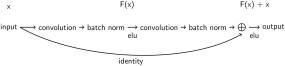
Residual block. Residual block used in the ResNet architecture and as described in original paper (He et al. [2015]; see Fig. 2) with identity shortcut option A, except using ELU instead of ReLU nonlinearities. See the section “Residual ConvNet for raw EEG signals” for explanation.

In addition to the shallow and deep ConvNets, we evaluated another network architecture: Residual networks (ResNets), a ConvNet architecture that recently won several benchmarks in the computer vision field [He et al., 2015]. ResNets typically have a very large number of layers and we wanted to investigate whether similar networks with more layers also result in good performance in EEG decoding. ResNets add the input of a convolutional layer to the output of the same layer, to the effect that the convolutional layer only has to learn to output a residual that changes the previous layers output (hence the name residual network). This allows ResNets to be successfully trained with a much larger number of layers than traditional convolutional networks [He et al., 2015]. Our residual blocks are the same as described in the original paper (Fig. 3).

Our ResNet used exponential linear unit activation functions [Clevert et al., 2016] throughout the network (same as the deep ConvNet) and also starts with a splitted temporal and spatial convolution (same as the deep and shallow ConvNets), followed by 14 residual blocks, mean pooling and a final softmax dense classification layer (for further details, see Supporting Information, Section A.3).

### ConvNet Training

In this section, we first give a definition of how ConvNets are trained in general. Second, we describe two ways of extracting training inputs and training labels from the EEG data, which result in a trialwise and a cropped training strategy.

#### Definition

To train a ConvNet, all parameters (all weights and biases) of the ConvNet are trained jointly. Formally, in our supervised classification setting, the ConvNet computes a function from input data to one real number per class, $f(X^{j};\theta ):{\mathbb{R}}^{E\cdot T}\to {\mathbb{R}}^{K}$, where *θ* are the parameters of the function, *E* the number of electrodes, *T* the number of timesteps, and *K* the number of possible output labels. To use ConvNets for classification, the output is typically transformed to conditional probabilities of a label *l_k_* given the input *X^j^* using the softmax function: $p(l_{k}|f(X^{j};\theta ))=\frac{\text{exp}(f_{k}(X^{j};\theta ))}{\sum_{m=1}^{K}\text{exp}(f_{m}(X^{j};\theta ))}$. In our case, as we train per subject, the softmax output gives us a subject‐specific conditional distribution over the *K* classes. Now we can train the entire ConvNet to assign high probabilities to the correct labels by minimizing the sum of the per‐example losses:

(1) $$\theta^{*}=\arg\underset{\theta }{\min}\sum_{j=1}^{N}loss\left(y^{j},p\left(l_{k}|f_{k}(X^{j};\theta )\right)\right),$$

where

(2) $$loss\left(y^{j},p\left(l_{k}|f_{k}(X^{j};\theta )\right)\right)=\sum_{k=1}^{K}-log\left(p\left(l_{k}|f_{k}(X^{j};\theta )\right)\right)\cdot \delta (y^{j}=l_{k})$$

is the negative log likelihood of the labels. As is common for training ConvNets, the parameters are optimized via mini‐batch stochastic gradient descent using analytical gradients computed via backpropagation (see LeCun et al. [2015] for an explanation in the context of ConvNets and the section “Optimization and early stopping” in this article for details on the optimizer used in this study).

This ConvNet training description is connected to our general EEG decoding definitions from the section “Definitions and Notation” as follows. The function that the ConvNet computes can be viewed as consisting of a feature extraction function and a classifier function: The feature extraction function $\phi (X^{j};\theta_{\phi })$ with parameters $\theta_{\phi }$ is computed by all layers up to the penultimate layer. The classification function $g\left(\phi (X^{j};\theta_{\phi }),\theta_{g}\right)$ with parameters *θ_g_*, which uses the output of the feature extraction function as input, is computed by the final classification layer. In this view, one key advantage of ConvNets becomes clear: With the joint optimization of both functions, a ConvNet learns both, a descriptive feature representation for the task and a discriminative classifier. This is especially useful with large datasets, where it is more likely that the ConvNet learns to extract useful features and does not just overfit to noise patterns. For EEG data, learning features can be especially valuable as there may be unknown discriminative features or at least discriminative features that are not used by more traditional feature extraction methods such as FBCSP.

#### Input and labels

In this study, we evaluated two ways of defining the input examples and target labels that the ConvNet is trained on. First, a trial‐wise strategy that uses whole trials as input and per‐trial labels as targets. Second, a cropped training strategy that uses crops, that is, sliding time windows within the trial as input and per‐crop labels as targets (where the label of a crop is identical to the label of the trial the crop was extracted from).

#### Trial‐wise training

The standard trial‐wise training strategy uses the whole duration of the trial and is therefore similar to how FBCSP is trained. For each trial, the trial signal is used as input and the corresponding trial label as target to train the ConvNet. We evaluated using trial epochs starting either at 500 ms before, directly at, or 500 ms after trial start cue, both for FBCSP and the ConvNets; 500 ms before trial start led to the best accuracies for the ConvNets, 500 ms after trial start to the best accuracies for FBCSP. Therefore, these settings (500 ms before for ConvNets, 500 ms after for FBCSP) were used in this study. This led to 288 training examples per subject for BCI competition IV dataset 2a and about 880 training examples per subject on the High‐Gamma Dataset after their respective train‐test split.

#### Cropped training

The cropped training strategy uses crops, that is, sliding input windows within the trial, which leads to many more training examples for the network than the trial‐wise training strategy. We adapted this strategy from convolutional neural networks for object recognition in images, where using multiple crops of the input image is a standard procedure to increase decoding accuracy (see, e.g., He et al. [2015] and Szegedy et al. [2015]).

In our study, we used crops of about 2 s as the input. We adopt a cropping approach, which leads to the largest possible number of crops by creating one crop per sample (by sample, we mean a timestep in our EEG trial time series). More formally, given an original trial $X^{j}\in {\mathbb{R}}^{E\cdot T}$ with *E* electrodes and *T* timesteps, we create a set of crops with crop size T′ as timeslices of the trial: $C^{j}=\{X_{1..E,t..t+T^{\prime }}^{j}|t\in 1..T-T\prime \}$. All of these T−T′ crops are new training data examples for our decoder and will get the same label *y^j^* as the original trial.

This aggressive cropping has the aim to force the ConvNet into using features that are present in all crops of the trial, as the ConvNet can no longer use the differences between crops and the global temporal structure of the features in the complete trial. We collected crops starting from 0.5 s before trial start (first crop from 0.5 s before to 1.5 s after trial start), with the last crop ending 4 s after the trial start (which coincides with the trial end, so the last crop starts 2 s before the trial and continues to the trial end). Overall, this resulted in 625 crops and therefore 625 label predictions per trial. The mean of these 625 predictions is used as the final prediction for the trial during the test phase. During training, we compute a loss for each prediction. Therefore, cropped training increases our training set size by a factor of 625, albeit with highly correlated training examples. As our crops are smaller than the trials, the ConvNet input size is also smaller (from about 1000 input samples to about 500 input samples for the 250 Hz sampling rate), while all other hyperparameters stay the same.

**Figure 4:**
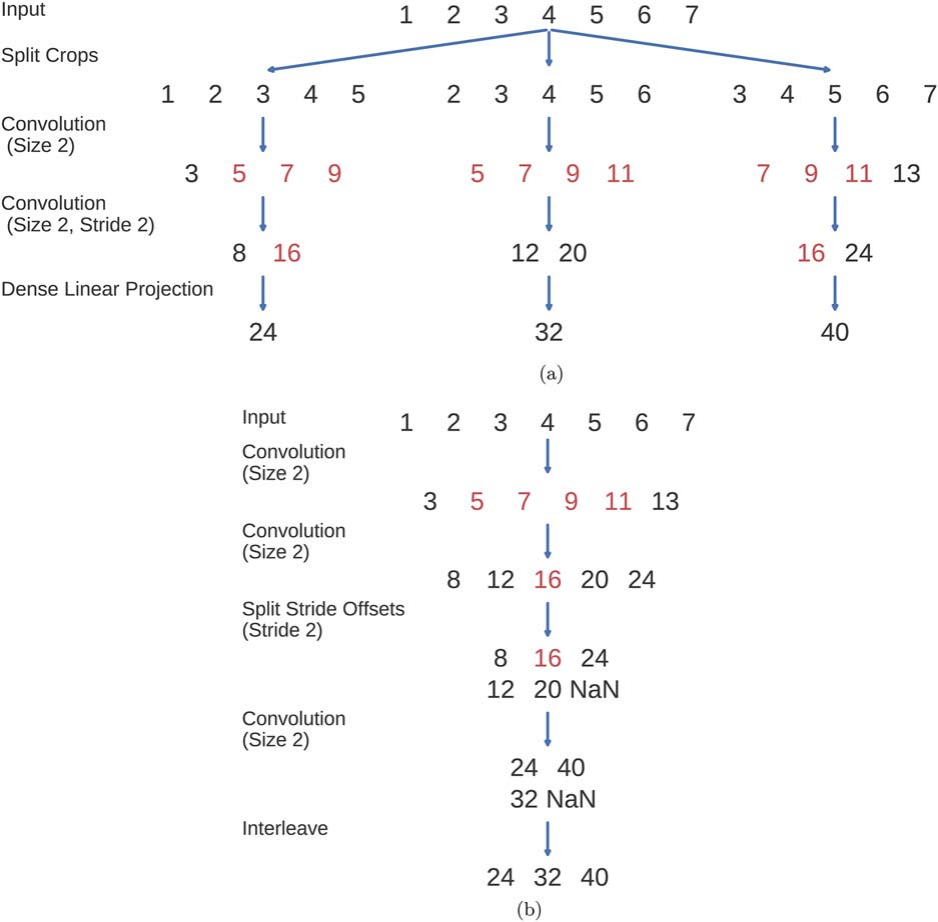
Multiple‐crop prediction used for cropped training. In this toy example, a trial with the sample values 1,2,3,4,5,6,7 is cut into three crops of length 5 and these crops are passed through a convolutional network with two convolutional layers and one dense layer. The convolutional layers both have kernel size 2, and the second one additionally uses a stride of 2. Filters for both layers and the final dense layer have values 1,1. Red indicates intermediate outputs that were computed multiple times in the naïve implementation. Note that both implementations result in the same final outputs. (a) Naïve implementation by first splitting the trial into crops and passing the crops through the ConvNet independently. (b) Optimized implementation, computing the outputs for each crop in a single forward pass. Strides in the original ConvNet are handled by separating intermediate results that correspond to different stride offsets, see the split stride offsets step. NaNs are only needed to pad all intermediate outputs to the same size and are removed in the end. The split stride step can simply be repeated in case of further layers with stride. We interleave the outputs only after the final predictions, also in the case of ConvNets with more layers. [Color figure can be viewed at http://wileyonlinelibrary.com]

To reduce the computational load from the increased training set size, we decoded a group of neighboring crops together and reused intermediate convolution outputs. This idea has been used in the same way to speed up ConvNets that make predictions for each pixel in an image [Giusti et al., 2013; Nasse et al., 2009; Sermanet et al., 2014; Shelhamer et al., 2016]. In a nutshell, this method works by providing the ConvNet with an input that contains several crops and computing the predictions for all crops in a single forward pass (see Fig. 4 for an explanation). This cropped training method leads to a new hyperparameter: the number of crops that are processed at the same time. The larger this number of crops, the larger the speedup one can get (upper bounded by the size of one crop, see Giusti et al. [2013] for a more detailed speedup analysis on images), at the cost of increased memory consumption. A larger number of crops that are processed at the same time during training also implies parameter updates from gradients computed on a larger number of crops from the same trial during mini‐batch stochastic gradient descent, with the risk of less stable training. However, we did not observe substantial accuracy decreases when enlarging the number of simultaneously processed crops (this stability was also observed for images [Shelhamer et al., 2016]) and in the final implementation, we processed about 500 crops in one pass, which corresponds to passing the ConvNet an input of about 1000 samples, twice the 500 samples of one crop. Note that this method only results in exactly the same predictions as the naïve method when using valid convolutions (i.e., no padding). For padded convolutions (which we use in the residual network described in the section “Residual ConvNet for raw EEG signals”), the method no longer results in the same predictions, so it cannot be used to speed up predictions for individual samples anymore. However, it can still be used if one is only interested in the average prediction for a trial as we are in this study.

To further regularize ConvNets trained with cropped training, we designed a new objective function, which penalizes discrepancies between predictions of neighboring crops. In this *tied sample loss function*, we added the cross‐entropy of two neighboring predictions to the usual loss of of negative log likelihood of the labels. So, denoting the prediction $p\left(l_{k}|f_{k}(X_{t..t+T\prime }^{j};\theta )\right)$ for crop $X_{t..t+T\prime }^{j}$ from time step *t* to t+T′ by $p_{f,k}(X_{t..t+T\prime }^{j})$, the loss now also depends on the prediction for the next crop $p_{f,k}(X_{t+1..t+T\prime +1}^{j})$ and changes from Eq. (2) to

(3) $$\begin{array}{c} loss\left(y^{j},p_{f,k}(X_{t..t+T\prime }^{j})\right)=\sum_{k=1}^{K}-log\left(p_{f,k}(X_{t..t+T\prime }^{j})\right)\cdot \delta (y^{j}=l_{k})\\ +\sum_{k=1}^{K}-log\left(p_{f,k}(X_{t..t+T\prime }^{j})\right)\cdot p_{f,k}(X_{t+1..t+T\prime +1}^{j}) \end{array}$$

This is meant to make the ConvNet focus on features which are stable for several neighboring input crops.

#### Architecture differences

The ConvNet architectures are identical for trial‐wise training and cropped training, except for the final classification layer. It has a larger weight size in the temporal dimension for the trial‐wise training than for the cropped training to process the larger temporal input length (final temporal weight length 9 vs 2 for the deep ConvNet and 69 vs 30 for the shallow ConvNet).

#### Optimization and early stopping

As optimization method, we used Adam [Kingma and Ba, 2014] together with a specific early stopping method, as this consistently yielded good accuracy in preliminary experiments on the training set. For details on Adam and our early stopping strategy, see Supporting Information, Section A.4.

### Visualization

#### Correlating input features and unit outputs: Network correlation maps

**Figure 5:**
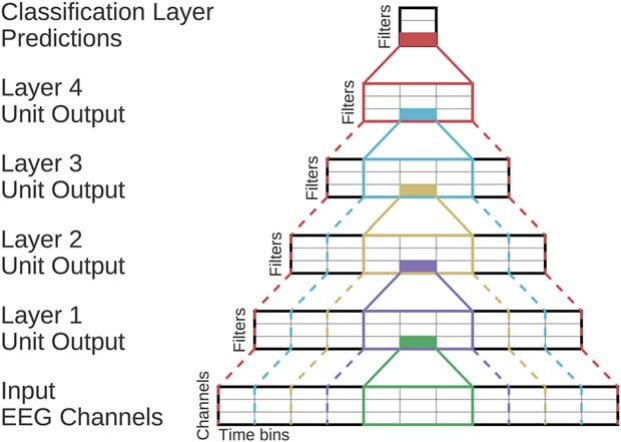
ConvNet Receptive Fields Schema. Showing the outputs, inputs, and receptive fields of one unit per layer. Colors indicate different units. Filled rectangles are individual units, and solid lines indicate their direct input from the layer before. Dashed lines indicate the corresponding receptive field in all previous layers including the original input layer. The receptive field of a unit contains all inputs that are used to compute the unit's output. The receptive fields get larger with increasing depth of the layer. Note that this is only a schema and exact dimensions are not meaningful in this figure. [Color figure can be viewed at http://wileyonlinelibrary.com]

As described in the Introduction, currently there is a great interest in understanding how ConvNets learn to solve different tasks. To this end, methods to visualize functional aspects of ConvNets can be helpful and the development of such methods is an active area of research. Here, we wanted to delineate what brain‐signal features the ConvNets used and in which layers they extracted these features. The most obvious restriction on possible features is that units in individual layers of the ConvNet can only extract features from samples that they have “seen,” that is, from their so‐called *receptive field* (Fig. 5). A way to further narrow down the possibly used features is to use domain‐specific prior knowledge and to investigate whether known class‐discriminative features are learned by the ConvNet. Then it is possible to compute a feature value for all receptive fields of all individual units for each of these class‐discriminative features and to measure how much this feature affects the unit output, for example, by computing the correlation between feature values and unit outputs.

In this spirit, we propose input‐feature unit‐output correlation maps as a method to visualize how networks learn spectral amplitude features. It is known that the amplitudes, for example of the alpha, beta and gamma bands, provide class‐discriminative information for motor tasks [Ball et al., 2008; Pfurtscheller, 1981; Pfurtscheller and Aranibar, 1979]. Therefore, we used the mean envelope values for several frequency bands as feature values. We correlated these values inside a receptive field of a unit, as a measure of its total spectral amplitude, with the corresponding unit outputs to gain insight into how much these amplitude features are used by the ConvNet. Positive or negative correlations that systematically deviate from those found in an untrained net imply that the ConvNet learned to create representations that contain more information about these features than before training.

A limitation of this approach is that it does not distinguish between correlation and causation (i.e., whether the change in envelope caused the change in the unit output, or whether another feature, itself correlated to the unit output, caused the change). Therefore, we propose a second visualization method, where we perturbed the amplitude of existing inputs and observed the change in predictions of the ConvNets. This complements the first visualization and we refer to this method as input‐perturbation network‐prediction correlation map. By using artificial perturbations of the data, they provide insights in whether changes in specific feature amplitudes cause the network to change its outputs. Details on the computation of both NCM methods are described in the following.

#### Input‐feature unit‐output correlation maps

**Figure 6:**
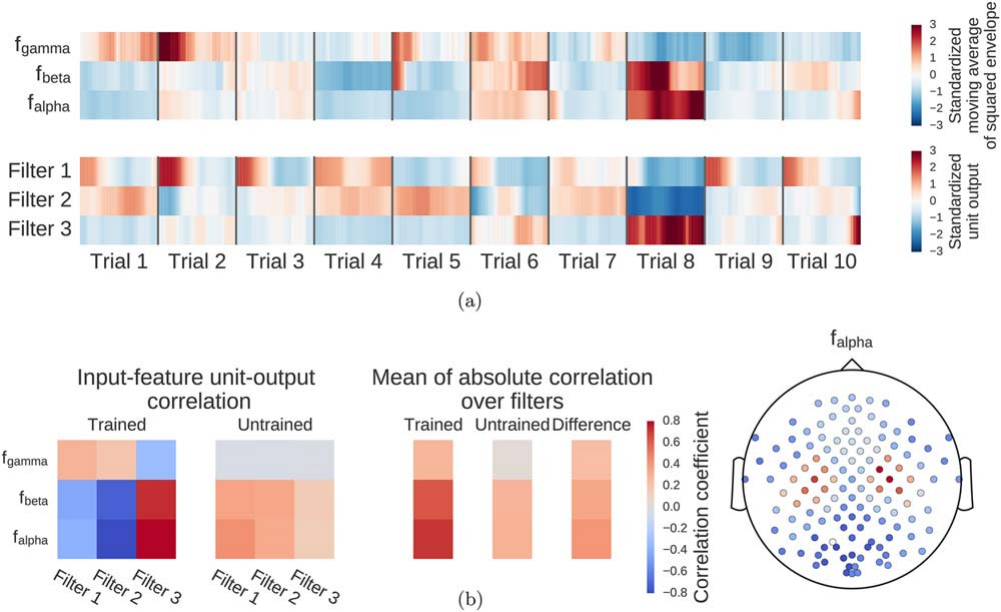
Computation overview for input‐feature unit‐output network correlation map. (a) Feature inputs and unit outputs for input‐feature unit‐output correlation map. Moving average of squared envelopes and unit outputs for 10 trials. Upper rows show mean squared envelopes over the receptive field for three frequency ranges in the alpha, beta, and gamma frequency band, standardized per frequency range. Lower rows show corresponding unit outputs for three filters, standardized per filter. All time series standardized for the visualization. (b) Input‐feature unit‐output correlations and corresponding scalp map for the alpha band. Left: Correlation coefficients between unit outputs of three filters and mean squared envelope values over the corresponding receptive field of the units for three frequency ranges in the alpha (7–13 Hz), beta (13–31 Hz), and gamma (71–91 Hz) frequency band. Results are shown for the trained and the untrained ConvNet and for one electrode. Middle: Mean of the absolute correlation coefficients over the three filters for the trained and the untrained ConvNet, and the difference between trained and untrained ConvNet. Right: An exemplary scalp map for correlations in the alpha band (7–13 Hz), where the color of each dot encodes the correlation difference between a trained and an untrained ConvNet for one electrode. Note localized positive effects above areas corresponding to the right and left sensorimotor hand/arm areas, indicating that activity in these areas has large absolute correlations with the predictions of the trained ConvNet. [Color figure can be viewed at http://wileyonlinelibrary.com]

The input‐feature unit‐output correlation maps visualize the frequency‐resolved correlation between unit outputs of the convolutional filters of the ConvNets and the power of all the samples in the receptive field of these units (Fig. 6).

To achieve this, we performed the following steps:

1. For each frequency band of interest, the signal was bandpass‐filtered to that frequency band and the envelope was computed.
2. For each frequency band of interest, the squared mean envelope for each receptive field of a given layer was computed. We did this by computing a moving window average of the squared envelope with the moving window size the same as the receptive field size (this was the input feature for which we then evaluated how much it affected the unit output).
3. Unit outputs of the given layer on the original signal were computed.
4. Linear correlations between the squared mean envelope values for all the frequency bands and the unit outputs for each convolutional filter were computed. These correlations should reflect whether a filter might compute an approximation of the squared mean envelope of all the samples in its receptive field.

**Figure 7:**
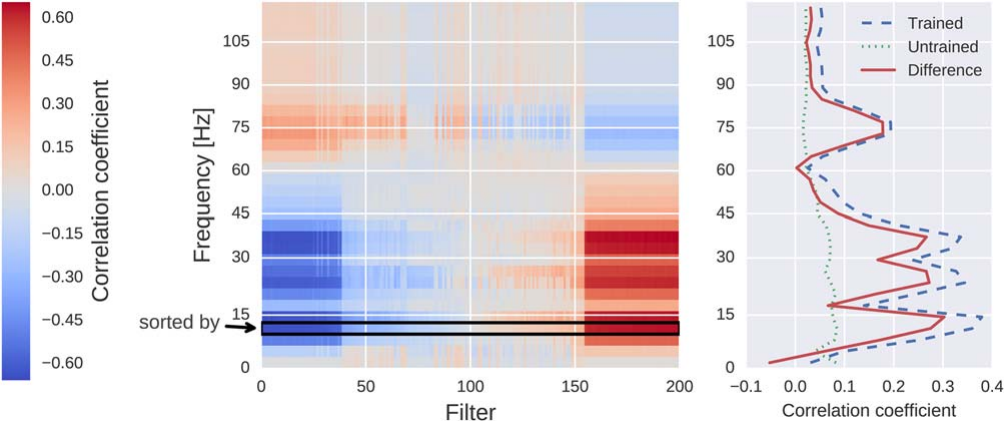
Correlation between the mean squared envelope feature and unit output for a single subject at one electrode position (FCC4h). Left: All correlations. Colors indicate the correlation between unit outputs per convolutional filter (*x*‐axis) and mean squared envelope in different frequency bands (*y*‐axis). Filters are sorted by their correlation to the 7–13 Hz envelope (outlined by the black rectangle). Note the large correlations/anticorrelations in the alpha/beta bands (7–31 Hz) and somewhat weaker correlations/anticorrelations in the gamma band (around 75 Hz). Right: mean absolute values across units of all convolutional filters for all correlation coefficients of the trained model, the untrained model and the difference between the trained and untrained model. Peaks in the alpha, beta, and gamma bands are clearly visible. [Color figure can be viewed at http://wileyonlinelibrary.com]

As we compute correlations after concatenating all samples of all trials, these correlations reflect both within‐trial and between‐trial effects. The proposed method could, however, be straightforwardly extended to disentangle these two sources. We computed the correlations for a filter bank ranging from 0 to 119 Hz. An example result for a single electrode and a single subject is shown in Figure 7.

To compute a single scalp plot for a frequency band, we computed the mean of the absolute correlations over all units for each convolutional filter and each electrode for that frequency band. To disentangle effects which are caused by the training of the network from those caused by the architecture, we computed the scalp plot for a trained and an untrained model. The scalp plot for a subject is then the scalp plot of the trained model minus the scalp plot of the untrained model (Fig. 6b). The group scalp plot is the mean of these differential scalp plots over all subjects.

To compare the resulting maps against scalp maps that simply result from class‐feature correlations, we also computed the linear correlation between mean squared envelope values and the one‐hot‐encoded classes, in the same way as before. First, for each trial, each sensor, and each frequency band, we constructed a vector of the moving window squared envelope values as before (with the moving window now the size of the receptive field of the last layer of the ConvNet). Second, for each trial and each class, we constructed a vector of either value 1 if the trial was of the given class or value 0 if it was of another class. The concatenated vectors then resulted in a time series with value 1 if the time point belonged to a given class and value 0 if it did not. Then we correlated the moving window squared envelope time series vectors with the class time series vectors, resulting in one correlation value per class, sensor, and frequency band combination. As in the other computations, we subtracted the correlations resulting from predictions of an untrained deep ConvNet.

A further question is whether the correlations could be a result of the unit outputs encoding the final class label. Such correlations could also result from using other discriminative features than the features we analyzed. To investigate this question, we correlated the unit outputs for each layer with the class labels. Here, we proceeded the same way as described in the previous paragraph, but correlated unit outputs directly with class labels. We then computed a single absolute correlation coefficient per layer in two ways: First, we computed the mean absolute correlation coefficient for all classes and all filters. These correlations should show how strongly the unit outputs encode the class labels on average across filters. Second, we computed the maximum absolute correlation coefficients for each class over all filters and then computed the mean of these maxima of the four classes. These correlations should show how strongly the unit outputs encode the class labels for the most “class‐informative” filters. Finally, for both versions and as in the other visualizations, we also computed the difference of these correlations between a trained and an untrained model. In summary, this approach allowed to show how unit‐output class correlations arise from layer to layer through the ConvNet.

#### Input‐perturbation network‐prediction correlation map

**Figure 8:**
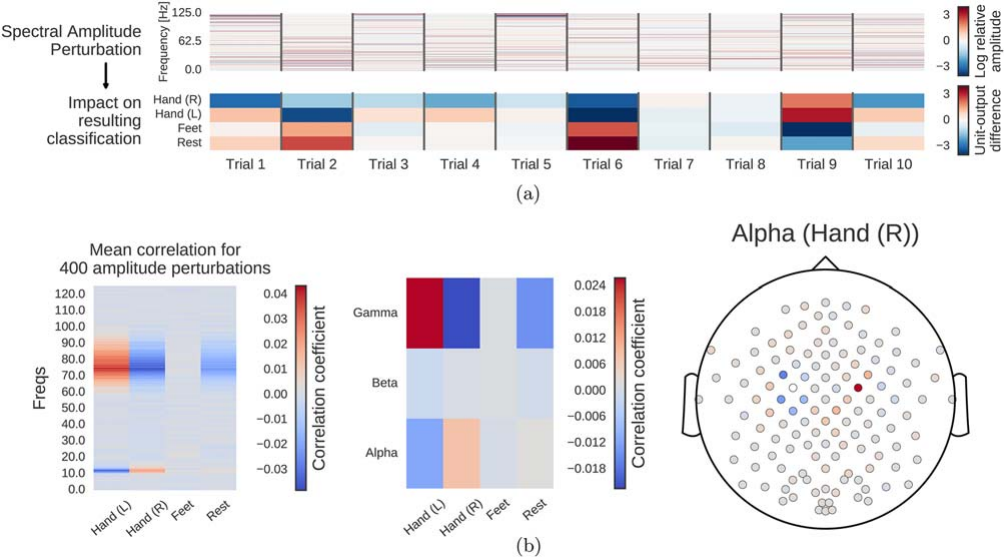
Computation overview for input‐perturbation network‐prediction correlation map. (a) Example spectral amplitude perturbation and resulting classification difference. Top: Spectral amplitude perturbation as used to perturb the trials. Bottom: unit‐output difference between unperturbed and perturbed trials for the classification layer units before the softmax. (b) Input‐perturbation network‐prediction correlations and corresponding network correlation scalp map for alpha band. Left: Correlation coefficients between spectral amplitude perturbations for all frequency bins and differences of the unit outputs for the four classes (differences between unperturbed and perturbed trials) for one electrode. Middle: Mean of the correlation coefficients over the the alpha (7–13 Hz), beta (13–31 Hz) and gamma (71–91 Hz) frequency ranges. Right: An exemplary scalp map for the alpha band, where the color of each dot encodes the correlation of amplitude changes at that electrode and the corresponding prediction changes of the ConvNet. Negative correlations on the left sensorimotor hand/arm areas show an amplitude decrease in these areas leads to a prediction increase for the Hand (R) class, whereas positive correlations on the right sensorimotor hand/arm areas show an amplitude decrease leads to a prediction decrease for the Hand (R) class. This complements the information from the input‐feature unit‐output network correlation map (Fig. 6b), which showed band power in these areas is strongly correlated with unit outputs in the penultimate layer. [Color figure can be viewed at http://wileyonlinelibrary.com]

To investigate the causal effect of changes in power on the deep ConvNet, we correlated changes in ConvNet predictions with changes in amplitudes by perturbing the original trial amplitudes (see Fig. 8 for an overview). Concretely, we transformed all training trials into the frequency domain by a Fourier transformation. Then we randomly perturbed the amplitudes by adding Gaussian noise (with mean 0 and variance 1) to them. The phases were kept unperturbed. After the perturbation, we retransformed to the time domain by the inverse Fourier transformation. We computed predictions of the deep ConvNet for these trials before and after the perturbation (predictions here refers to outputs of the ConvNet directly before the softmax activation). We repeated this procedure with 400 perturbations sampled from aforementioned Gaussian distribution and then correlated the change in input amplitudes (i.e., the perturbation/noise we added) with the change in the ConvNet predictions. To ensure that the effects of our perturbations reflect the behavior of the ConvNet on realistic data, we also checked that the perturbed input does not cause the ConvNet to misclassify the trials (as can easily happen even from small perturbations, see Szegedy et al. [2014]). For that, we computed accuracies on the perturbed trials. For all perturbations of the training sets of all subjects, accuracies stayed above 99.5% of the accuracies achieved with the unperturbed data.

### Datasets and Preprocessing

We first evaluated decoding accuracies on two EEG datasets, a smaller public dataset (BCI competition IV dataset 2a [Brunner et al., 2008]) for comparing to previously published accuracies and a larger new dataset acquired in our lab for evaluating the decoding methods with a larger number of training trials (∼880 trials per subject, compared to 288 trials in the public set). We call this dataset High Gamma Dataset (HGD), as it is especially well‐suited for extracting information from the higher frequencies; see Supporting Information, Section A.6. To investigate whether our main results also hold on other datasets, we compared the ConvNet decoding accuracies with FBCSP on two additional datasets: The BCI Competition IV 2b dataset, a 3‐electrode two‐class EEG motor‐imagery dataset, and the Mixed Imagery Dataset (MID), a 64‐electrode four‐class dataset with both two motor and two non‐motor imagery classes (right hand movement, feet movement, mental rotation and word generation). For details on the datasets, see Supporting Information, Section A.6.

#### EEG preprocessing and evaluating different frequency bands

We only minimally preprocessed the datasets to allow the ConvNets to learn any further transformations themselves. In addition to the full‐bandwidth (0–*f_end_*‐Hz) dataset, we analyzed data high‐pass filtered above 4 Hz (which we call 4–*f_end_*‐Hz dataset) to minimize artifacts, which need to be considered in brain‐signal decoding for brain‐computer interfaces. Filtering was done with a causal third‐order Butterworth filter. We included the 4–*f_end_*‐Hz dataset as the highpass filter should make it less probable that either the networks or FBCSP would use class‐discriminative eye movement artifacts to decode the behavior classes, as eye movements generate most power in such low frequencies [Gratton, 1998]. We included this analysis as for the BCI competition datasets special care to avoid decoding eye‐related signals was requested from the publishers of the datasets [Brunner et al., 2008] and a highpass filter was one of the suggested methods to remove eye artefacts; indeed this was the method the winners of the competition used [Ang et al., 2008]. For details on other preprocessing steps, see Supporting Information, Section A.7.

### Statistics

We used Wilcoxon signed‐rank tests to check for statistical significance of the mean difference of accuracies between decoding methods [Wilcoxon, 1945]. We handled ties by using the average rank of all tied data points and zero differences by assigning half of the rank‐sum of these zero differences to the positive rank‐sums and the other half to the negative rank‐sums. In case of a noninteger test‐statistic value caused by ties or zeros, we rounded our test‐statistic to the next larger integer, resulting in a more conservative estimate.

To correct for multiple tests, we additionally performed a false‐discovery‐rate correction at α=0.05 for all comparisons involving ConvNet accuracies using the Benjamini–Hochberg procedure [Benjamini and Hochberg, 1995].

## RESULTS

### Validation of FBCSP Baseline

#### Result 1: FBCSP baseline reached same results as previously reported in the literature

As a first step before moving to the evaluation of ConvNet decoding, we validated our FBCSP implementation, as this was the baseline we compared the ConvNets results against. To validate our FBCSP implementation, we compared its accuracies to those published in the literature for the BCI competition IV dataset 2a [Sakhavi et al., 2015]. Using the same 0.5–2.5 s (relative to trial onset) time window, we reached an accuracy of 67.6%, statistically not significantly different from theirs (67.0%, *P* = 0.73, Wilcoxon signed‐rank test). Note, however, that we used the full trial window for later experiments with convolutional networks, that is, from 0.5 to 4 s. This yielded a slightly better accuracy of 67.8%, which was still not statistically significantly different from the original results on the 0.5–2.5 s window (*P* = 0.73). For all later comparisons, we use the 0.5–4 s time window on all datasets.

### Architectures and Design Choices

#### Result 2: ConvNets reached FBCSP accuracies

**Figure 9:**
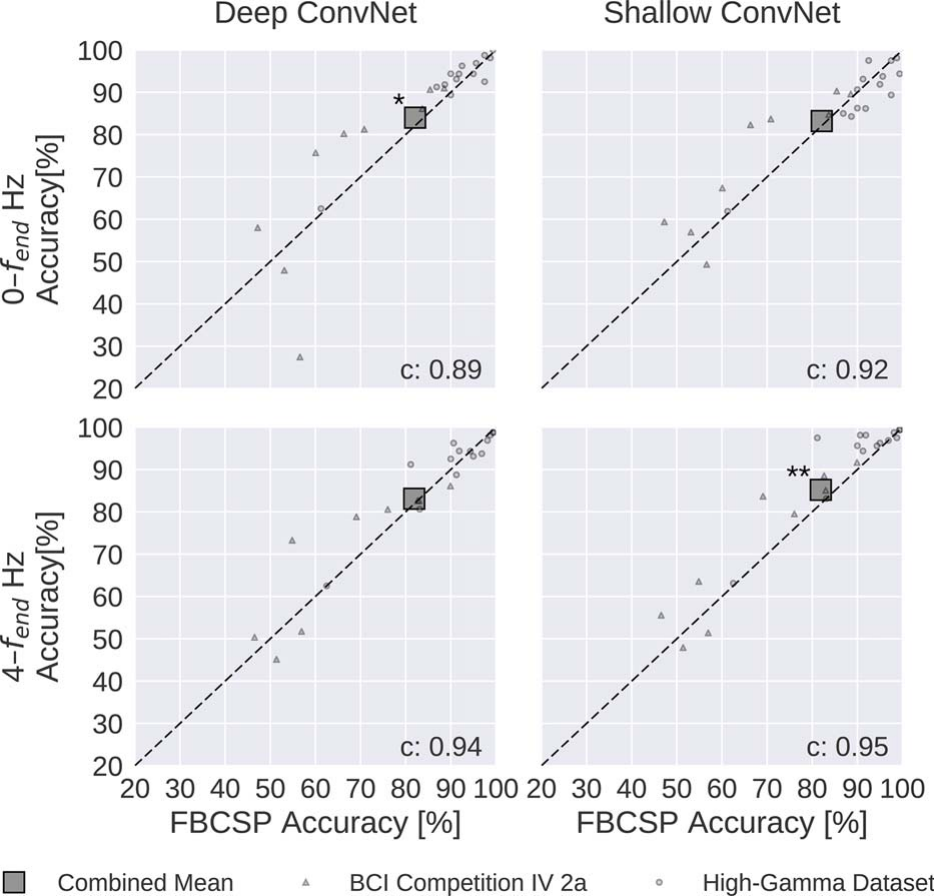
FBCSP versus ConvNet decoding accuracies. Each small marker represents the accuracy of one subject, the large square markers represent average accuracies across all subjects of both datasets. Markers above the dashed line indicate experiments where ConvNets performed better than FBCSP and opposite for markers below the dashed line. Stars indicate statistically significant differences between FBCSP and ConvNets (Wilcoxon signed‐rank test, *P* < 0.05: *, *P* < 0.01: **, *P* < 0.001=***). Bottom left of every plot: linear correlation coefficient between FBCSP and ConvNet decoding accuracies. Mean accuracies were very similar for ConvNets and FBCSP, the (small) statistically significant differences were in direction of the ConvNets. [Color figure can be viewed at http://wileyonlinelibrary.com]

Both the deep the shallow ConvNets, with appropriate design choices (see Result 5), reached similar accuracies as FBCSP‐based decoding, with small but statistically significant advantages for the ConvNets in some settings. For the mean of all subjects of both datasets, accuracies of the shallow ConvNet on 0–*f_end_* Hz and for the deep ConvNet on 4–*f_end_* Hz were not statistically significantly different from FBCSP (Fig. 9 and Table 2). The deep ConvNet on 0–*f_end_* Hz and the shallow ConvNet on 4—*f_end_* Hz reached slightly higher (1.9% and 3.3% higher, respectively) accuracies that were also statistically significantly different (*P*  <  0.05, Wilcoxon signed‐rank test). Note that all results in this section were obtained with cropped training, for a comparison of cropped and trial‐wise training, see the section “Training Strategy.” Note that all *P* values below 0.01 in this study remain significant when controlled with false‐discovery‐rate correction at α=0.05 across all tests involving ConvNet accuracies.

**Table 2:** Decoding accuracy of the FBCSP baseline and of the deep and shallow ConvNets

Dataset	Frequency range [Hz]	FBCSP	Deep ConvNet	Shallow ConvNet	Hybrid ConvNet	Residual ConvNet
BCIC IV 2a	0–38	68.0	+2.9	+5.7*	+3.6	−0.3
BCIC IV 2a	4–38	67.8	+2.3	+4.1	−1.6	−7.0*
HGD	0–125	91.2	+1.3	−1.9	+0.6	−2.3*
HGD	4–125	90.9	+0.5	+3.0*	+1.5	−1.1
Combined	0–*f_*end*_*	82.1	+1.9*	+1.1	+1.8	−1.1
Combined	4–*f_*end*_*	81.9	+1.2	+3.4**	+0.3	−3.5*

FBCSP decoding accuracies and difference of deep and shallow ConvNet accuracies to FBCSP results are given in percentage. BCIC IV 2a: BCI competition IV dataset 2a. HGD: High‐Gamma Dataset. Frequency range is in Hertz. Stars indicate statistically significant differences (*P* values from Wilcoxon signed‐rank test, *: *P* < 0.05, **: *P* < 0.01, no *P* values were below 0.001). Note that all *P* values below 0.01 in this study remain significant when controlled with false‐discovery‐rate correction at α=0.05 across all tests involving ConvNet accuracies.

#### Result 3: Confusion matrices for all decoding approaches were similar

**Figure 10:**
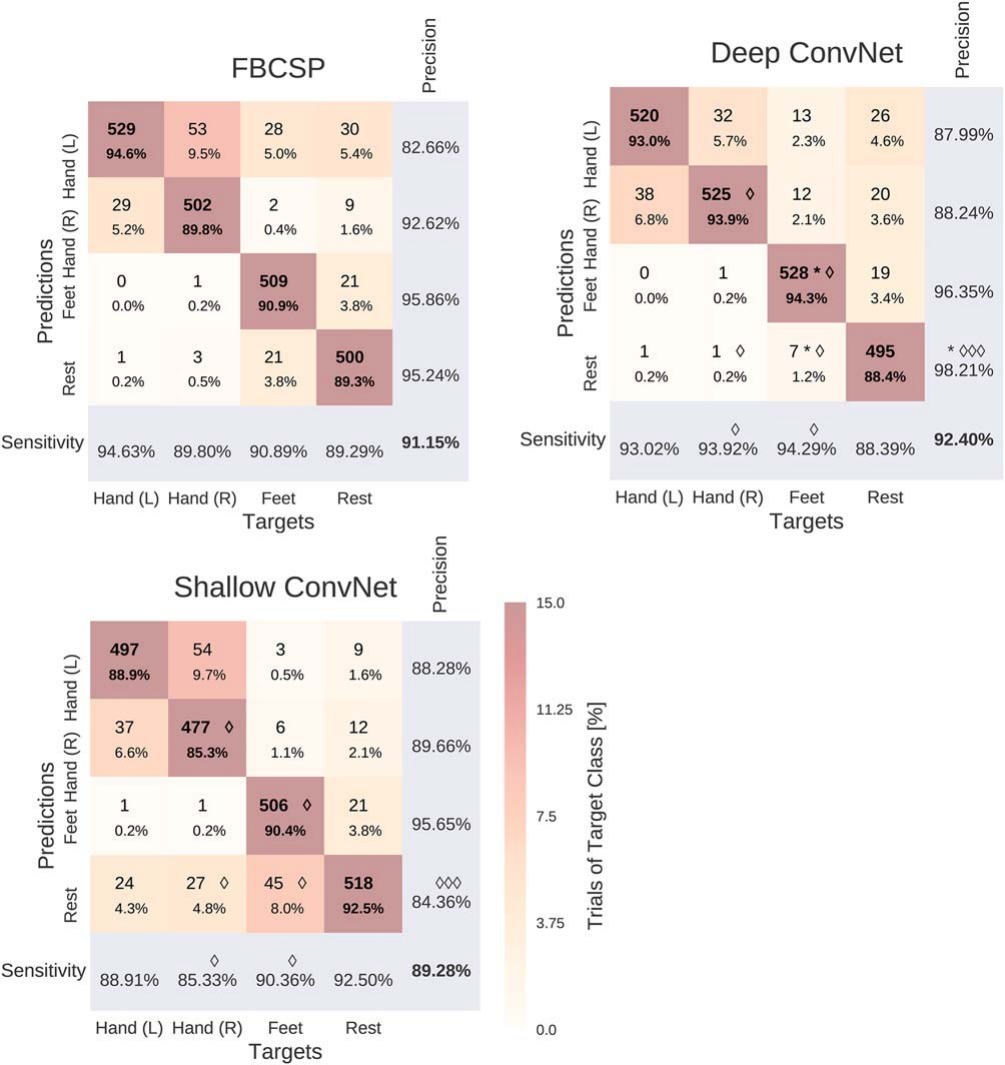
Confusion matrices for FBCSP‐ and ConvNet‐based decoding. Results are shown for the High‐Gamma Dataset, on 0–*f_end_* Hz. Each entry of row r and column c for upper‐left 4×4‐square: Number of trials of target r predicted as class c (also written in percent of all trials). Bold diagonal corresponds to correctly predicted trials of the different classes. Percentages and colors indicate fraction of trials in this cell from all trials of the corresponding column (i.e., from all trials of the corresponding target class). The lower‐right value corresponds to overall accuracy. Bottom row corresponds to sensitivity defined as the number of trials correctly predicted for class c/number of trials for class c. Rightmost column corresponds to precision defined as the number of trials correctly predicted for class r/number of trials predicted as class r. Stars indicate statistically significantly different values of ConvNet decoding from FBCSP, diamonds indicate statistically significantly different values between the shallow and deep ConvNets. *P*<0.05: ⋄/*, *P*<0.01: ⋄⋄/**, *P*<0.001: ⋄⋄⋄/***, Wilcoxon signed‐rank test. [Color figure can be viewed at http://wileyonlinelibrary.com]

Confusion matrices for the High‐Gamma Dataset on 0–*f_end_* Hz were very similar for FBCSP and both ConvNets (Fig. 10). The majority of all mistakes were due to discriminating between Hand (L)/Hand (R) and Feet/Rest, see Table 3. Seven entries of the confusion matrix had a statistically significant difference (*P*  < 0.05, Wilcoxon signed‐rank test) between the deep and the shallow ConvNet, in all of them the deep ConvNet performed better. Only two differences between the deep ConvNet and FBCSP were statistically significant (*P*  < 0.05), none for the shallow ConvNet and FBCSP. Confusion matrices for the BCI competition IV dataset 2a showed a larger variability and hence a less consistent pattern, possibly because of the much smaller number of trials.

**Table 3:** Decoding errors between class pairs

	Hand (L) Hand (R)	Hand (L) Feet	Hand (L) Rest	Hand (R) Feet	Hand (R) Rest	Feet Rest
FBCSP	82	28	31	3	12	42
Deep	70	13	27	13	21	26
Shallow	99	3	34	5	37	73

Results for the High‐Gamma Dataset. Number of trials where one class was mistaken for the other for each decoding method, summed per class pair. The largest number of errors was between Hand (L) and Hand (R) for all three decoding methods, the second largest between Feet and Rest (on average across the three decoding methods). Together, these two class pairs accounted for more than 50% of all errors for all three decoding methods. In contrast, Hand (L and R) and Feet had a small number of errors irrespective of the decoding method used.

#### Result 4: Hybrid ConvNets performed slightly, but statistically insignificantly, worse than deep ConvNets

The hybrid ConvNet performed similar, but slightly worse than the deep ConvNet, that is, 83.8% vs 84.0% (*P*  > 0.5, Wilcoxon signed‐rank test) on the 0–*f_end_*‐Hz dataset, 82.1% vs 83.1% (*P*  > 0.9) on the 4–*f_end_*‐Hz dataset. In both cases, the hybrid ConvNet's accuracy was also not statistically significantly different from FBCSP (83.8% vs 82.1%, *P*  >  0.4 on 0–*f_end_* Hz, 82.1% vs 81.9%, *P*  > 0.7 on 4–*f_end_* Hz).

#### Result 5: ConvNet design choices substantially affected decoding accuracies

In the following, results for all design choices are reported for all subjects from both datasets. For an overview of the different design choices investigated, and the motivation behind these choices, we refer to section “Design choices for deep and shallow ConvNet.”

**Figure 11:**
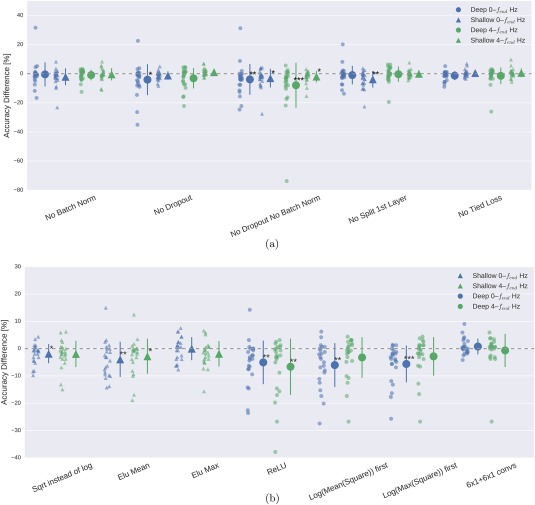
Impact of ConvNet design choices on decoding accuracy. Accuracy differences of baseline and design choices on *x*‐axis for the 0–*f_end_*‐Hz and 4–*f_end_*‐Hz datasets. Each small marker represents accuracy difference for one subject, and each larger marker represents mean accuracy difference across all subjects of both datasets. Bars: standard error of the differences across subjects. Stars indicate statistically significant differences to baseline (Wilcoxon signed‐rank test, *P* < 0.05: *, *P* < 0.01: **, *P* < 0.001=***). (a) Impact of design choices applicable to both ConvNets. Shown are the effects from the removal of one aspect from the architecture on decoding accuracies. All statistically significant differences were accuracy decreases. Notably, there was a clear negative effect of removing both dropout and batch normalization, seen in both ConvNets' accuracies and for both frequency ranges. (b) Impact of different types of nonlinearities, pooling modes and filter sizes. Results are given independently for the deep ConvNet and the shallow ConvNet. As before, all statistically significant differences were from accuracy decreases. Notably, replacing ELU by ReLU as nonlinearity led to decreases on both frequency ranges, which were both statistically significant. [Color figure can be viewed at http://wileyonlinelibrary.com]

Batch normalization and dropout significantly increased accuracies. This became especially clear when omitting both simultaneously (Fig. 11a). Batch normalization provided a larger accuracy increase for the shallow ConvNet, whereas dropout provided a larger increase for the deep ConvNet. For both networks and for both frequency bands, the only statistically significant accuracy differences were accuracy decreases after removing dropout for the deep ConvNet on 0–*f_end_*‐Hz data or removing batch normalization and dropout for both networks and frequency ranges (*P*  < 0.05, Wilcoxon signed‐rank test). Usage of tied loss did not affect the accuracies very much, never yielding statistically significant differences (*P*  > 0.05). Splitting the first layer into two convolutions had the strongest accuracy increase on the 0–*f_end_*‐Hz data for the shallow ConvNet, where it is also the only statistically significant difference (*P*  < 0.01).

For the deep ConvNet, using ReLU instead of ELU as nonlinearity in all layers worsened performance (*P*  < 0.01, see Fig. 11b on the right side). Replacing the 10 × 1 convolutions by 6 × 1 + 6 × 1 convolutions did not statistically significantly affect the performance (*P*  > 0.4).

#### Result 6: Recent deep learning advances substantially increased accuracies

**Figure 12:**
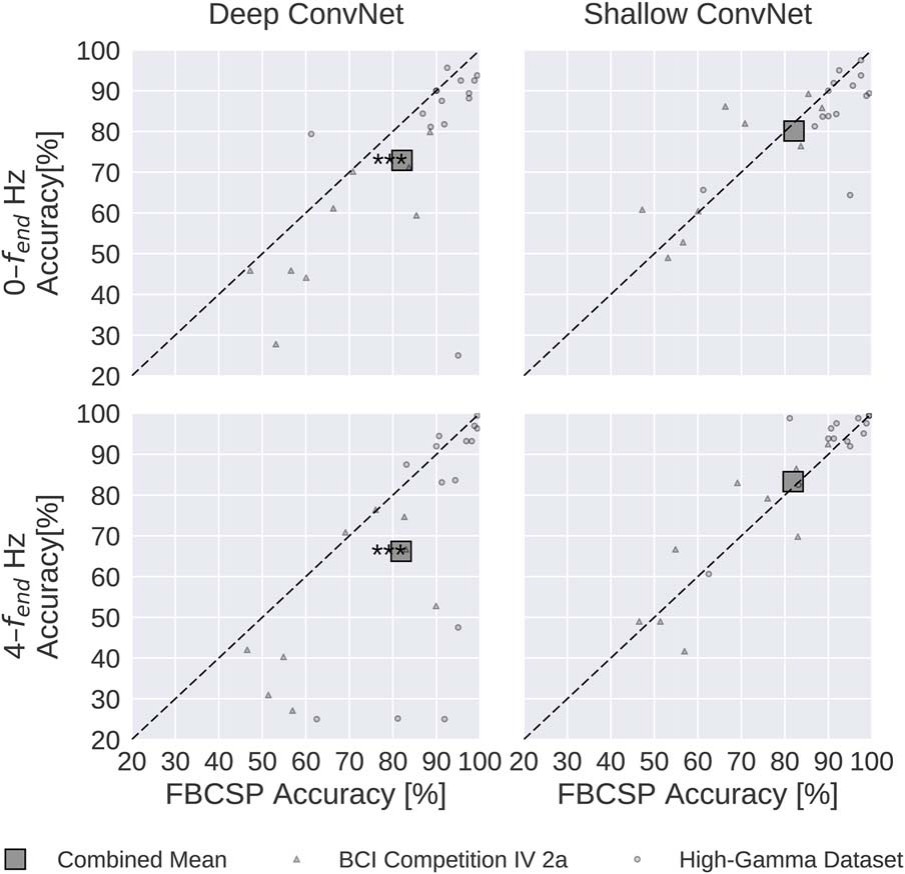
Impact of recent advances on overall decoding accuracies. Accuracies without batch normalization, dropout and ELUs. All conventions as in Figure 9. In contrast to the results on Figure 9, the deep ConvNet without implementation of these recent methodological advances performed worse than FBCSP; the difference was statistically significant for both frequency ranges. [Color figure can be viewed at http://wileyonlinelibrary.com]

Figure 12 clearly shows that only recent advances in deep learning methods (by which we mean the combination of batch normalization, dropout and ELUs) allowed our deep ConvNet to be competitive with FBCSP. Without these recent advances, the deep ConvNet had statistically significantly worse accuracies than FBCSP for both 0–*f_end_*‐Hz and 4–*f_end_*‐Hz data (*P*  < 0.001, Wilcoxon signed‐rank test). The shallow ConvNet was less strongly affected, with no statistically significant accuracy difference to FBCSP (*P*  > 0.2).

#### Result 7: Residual network performed worse than deep ConvNet

Residual networks had consistently worse accuracies than the deep ConvNet as seen in Table 4. All accuracies were lower and the difference was statistically significant for both frequency ranges on the combined dataset.

**Table 4:** Decoding accuracies residual networks and difference to deep ConvNets

Dataset	Frequency range [Hz]	Accuracy	Difference to deep	*P* value
BCIC IV 2a	0–38	67.7	−3.2	0.13
BCIC IV 2a	4–38	60.8	−9.3	0.004**
HGD	0–125	88.9	−3.5	0.020*
HGD	4–125	89.8	−1.6	0.54
Combined	0–*f_*end*_*	80.6	−3.4	0.004**
Combined	4–*f_*end*_*	78.5	−4.9	0.01*

BCIC IV 2a: BCI competition IV dataset 2a. HGD: High‐Gamma Dataset. Accuracy is mean accuracy in percentage. *P* value from Wilcoxon signed‐rank test for the statistical significance of the differences to the deep ConvNet (cropped training). Accuracies were always slightly worse than for the deep ConvNet, statistically significantly different for both frequency ranges on the combined dataset.

### Training Strategy

#### Result 8: Cropped training strategy improved deep ConvNet on higher frequencies

**Figure 13:**
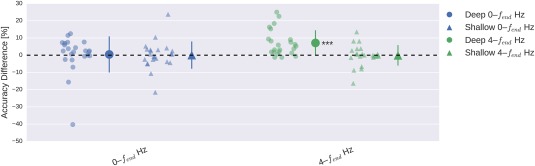
Impact of training strategy (cropped vs trial‐wise training) on accuracy. Accuracy difference for both frequency ranges and both ConvNets when using cropped training instead of trial‐wise training. Other conventions as in Figure 11. Cropped training led to better accuracies for almost all subjects for the deep ConvNet on the 4–*f_end_*‐Hz frequency range. [Color figure can be viewed at http://wileyonlinelibrary.com]

Cropped training increased accuracies statistically significantly for the deep ConvNet on the 4–*f_end_*‐Hz data (*P*<1e^−5^, Wilcoxon signed‐rank test; Fig. 13). In all other settings (0–*f_end_*‐Hz data, shallow ConvNet), the accuracy differences were not statistically significant ( P>0.1) and showed a lot of variation between subjects.

#### Result 9: Training ConvNets took substantially longer than FBCSP

FBCSP was substantially faster to train than the ConvNets with cropped training, by a factor of 27–45 on the BCI competition IV dataset 2a and a factor of 5–9 on the High‐Gamma Dataset (Table 5). Training times are end‐to‐end, that is, include the loading and preprocessing of the data. These times are only meant to give a rough estimate of the training times as there were differences in the computing environment between ConvNets training and FBCSP training. Most importantly, FBCSP was trained on CPU, while the networks were trained on GPUs (Supp. A.8). Longer relative training times for FBCSP on the High‐Gamma Dataset can be explained by the larger number of frequency bands we use on the High‐Gamma Dataset. Online application of the trained ConvNets does not suffer from the same speed disadvantage compared to FBCSP; the fast prediction speed of trained ConvNets make them well suited for decoding in real‐time BCI applications6.

**Table 5:** Training times

Dataset	FBCSP	std	Deep ConvNet	std	Shallow ConvNet	Std
BCIC IV 2a	00:00:33	<00:00:01	00:24:46	00:06:01	00:15:07	00:02:54
HGD	00:06:40	00:00:54	1:00:40	00:27:43	00:34:25	00:16:40

Mean times across subjects given in Hours:Minutes:Seconds. BCIC IV 2a: BCI competition IV dataset 2a. HGD: High‐Gamma Dataset. Std is standard deviation across subjects. ConvNets take substantially longer to train than FBCSP, especially the deep ConvNet.

**Table 6:** Decoding results for additional datasets

Dataset	Metric	FBCSP	Deep ConvNet	Shallow ConvNet
BCIC IV 2b	Kappa	0.599	−0.001	+0.300
MID	Accuracy [%]	71.2	+1.0	−3.5

For both datasets, the respective metric, FBCSP decoding performance, and percent differences of deep and shallow ConvNet decoding performance are given. BCIC IV 2b: BCI competition IV dataset 2b. MID: Mixed‐Imagery Dataset.

### Additional Datasets

#### Result 10: ConvNets reach accuracies in the same range as FBCSP on additional datasets

On the two additional datasets, the BCI competition IV dataset 2b and the Mixed‐Imagery Dataset (MID), ConvNets again reached accuracies in a very similar range as FBCSP. For BCI competition IV dataset 2b, deep ConvNets reached a mean kappa value of 0.598, almost identical to the FBCSP competition results (0.599), and shallow ConvNets reached a slightly better kappa value of 0.629. Both ConvNet results were not statistically significantly different from FBCSP (*P*  > 0.3). For the Mixed‐Imagery Dataset, shallow ConvNets reached a mean accuracy of 67.7%, slightly worse than FBCSP with 71.2%, whereas deep ConvNets reached slightly better accuracies with 72.2%.

### Visualization

#### Result 11: Band power topographies show event‐related “desynchronization/synchronization” typical for motor tasks

**Figure 14:**
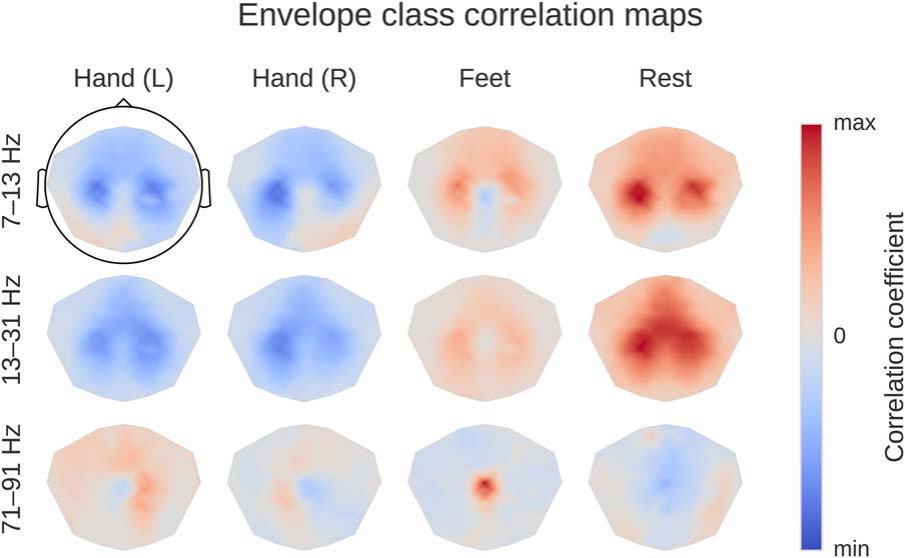
Envelope‐class correlations for alpha, beta, and gamma bands for all classes. Average over subjects from the High‐Gamma Dataset. Colormaps are scaled per frequency band/row. This is a ConvNet‐independent visualization, for an explanation of the computation see the section “Input‐feature unit‐output correlation maps.” Scalp plots show spatial distributions of class‐related spectral amplitude changes well in line with the literature. [Color figure can be viewed at http://wileyonlinelibrary.com]

Before moving to ConvNet visualization, we examined the spectral amplitude changes associated with the different movement classes in the alpha, beta, and gamma frequency bands, finding the expected overall scalp topographies (Fig. 14). For example, for the alpha (7–13 Hz) frequency band, there was a class‐related power decrease (anticorrelation in the class‐envelope correlations) in the left and right pericentral regions with respect to the hand classes, stronger contralaterally to the side of the hand movement, that is, the regions with pronounced power decreases lie around the primary sensorimotor hand representation areas. For the feet class, there was a power decrease located around the vertex, that is, approximately above the primary motor foot area. As expected, opposite changes (power increases) with a similar topography were visible for the gamma band (71–91 Hz).

#### Result 12: Input‐feature unit‐output correlation maps show learning progression through the ConvNet layers

**Figure 15:**
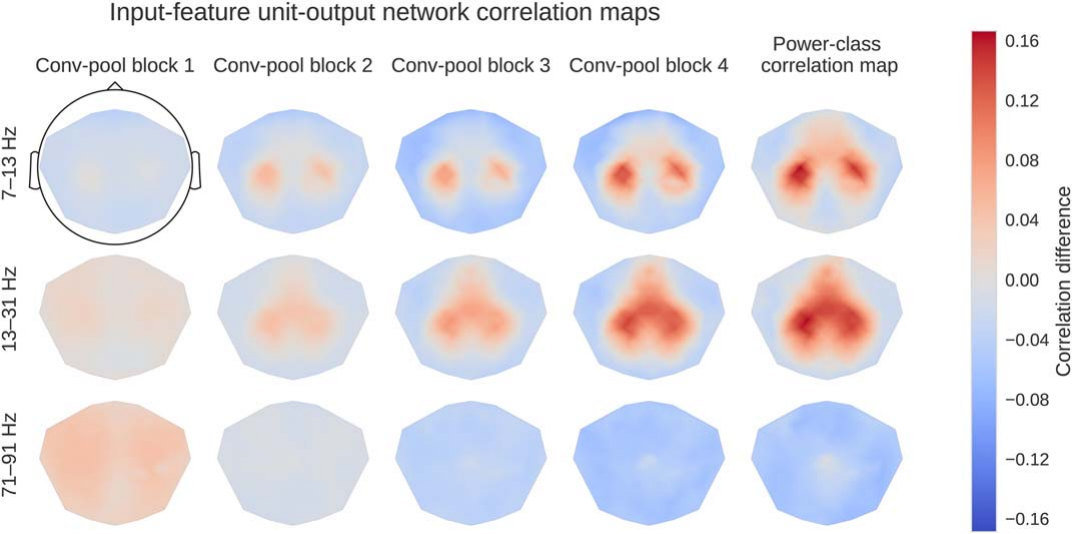
Power input‐feature unit‐output network correlation maps for all conv‐pool blocks of the deep ConvNet. Correlation difference indicates the difference of correlation coefficients obtained with the trained and untrained model for each electrode respectively and is visualized as a topographic scalp plot. For details, see the section “Input‐feature unit‐output correlation maps.” Rightmost column shows the correlation between the envelope of the EEG signals in each of the three analyzed frequency bands and the four classes. All colormaps are on the same scale. Notably, the absolute values of the correlation differences became larger in the deeper layers and converged to patterns that were very similar to those obtained from the power–class correlations. [Color figure can be viewed at http://wileyonlinelibrary.com]

**Figure 16:**
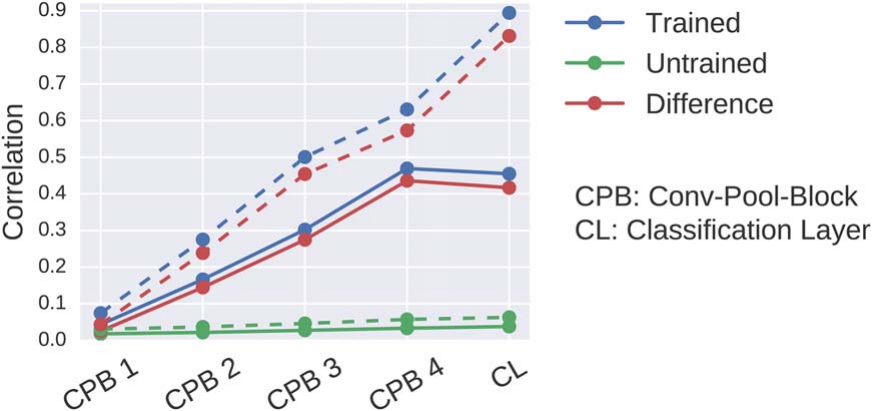
Absolute correlations between unit outputs and class labels. Each dot represents absolute correlation coefficients for one layer of the deep ConvNet. Solid lines indicate result of taking mean over absolute correlation coefficients between classes and filters. Dashed lines indicate result of first taking the maximum absolute correlation coefficient per class (maximum over filters) and then the mean over classes. Absolute correlations increased almost linearly with increasing depth of the layer. [Color figure can be viewed at http://wileyonlinelibrary.com]

We used our input‐feature unit‐output correlation mapping technique to examine the question how correlations between EEG power and the behavioral classes are learnt by the network. Figure 15 shows the input‐feature unit‐output correlation maps for all four conv‐pooling‐blocks of the deep ConvNet, for the group of subjects of the High‐Gamma Dataset. As a comparison, the figure also contains the correlation between the power and the classes themselves as described in the section “Input‐feature unit‐output correlation maps”. The differences of the absolute correlations show which regions were more correlated with the unit outputs of the trained ConvNet than with the unit outputs of the untrained ConvNet; these correlations are naturally undirected. Overall, the input‐feature unit‐output correlation maps became more similar to the power–class correlation maps with increasing layer depth. This gradual progression was also reflected in an increasing correlation of the unit outputs with the class labels with increasing depth of the layer (Fig. 16).

#### Result 13: Input‐perturbation network‐prediction correlation maps show causal effect of spatially localized band power features on ConvNet predictions

We show three visualizations extracted from input‐perturbation network‐prediction correlations, the first two to show the frequency profile of the causal effects, the third to show their topography.

**Figure 17:**
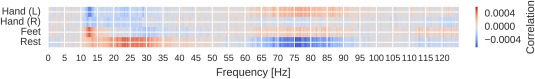
Input‐perturbation network‐prediction correlations for all frequencies for the deep ConvNet, per class. Plausible correlations, for example, rest positively, other classes negatively correlated with the amplitude changes in frequency range from 20 to 30 Hz. [Color figure can be viewed at http://wileyonlinelibrary.com]

Thus, first, we computed the mean across electrodes for each class separately to show correlations between classes and frequency bands. We see plausible results, for example, for the rest class, positive correlations in the alpha and beta bands and negative correlations in the gamma band (Fig. 17).

**Figure 18:**
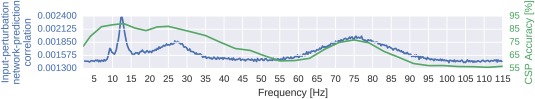
Absolute input‐perturbation network‐prediction correlation frequency profile for the deep ConvNet. Mean absolute correlation value across classes. CSP binary decoding accuracies for different frequency bands for comparison, averaged across subjects and class pairs. Peaks in alpha, beta, and gamma band for input‐perturbation network‐prediction correlations and CSP accuracies. [Color figure can be viewed at http://wileyonlinelibrary.com]

Then, second, by taking the mean of the absolute values both over all classes and electrodes, we computed a general frequency profile. This showed clear peaks in the alpha, beta, and gamma bands (Fig. 18). Similar peaks were seen in the means of the CSP binary decoding accuracies for the same frequency range.

**Figure 19:**
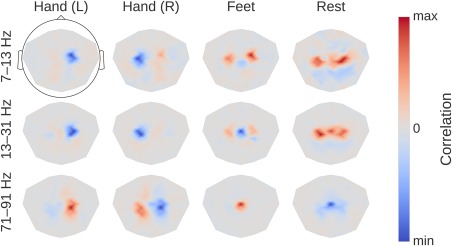
Input‐perturbation network‐prediction correlation maps for the deep ConvNet. Correlation of class predictions and amplitude changes. Averaged over all subjects of the High‐Gamma Dataset. Colormaps are scaled per scalp plot. Plausible scalp maps for all frequency bands, for example, contralateral positive correlations for the hand classes in the gamma band. [Color figure can be viewed at http://wileyonlinelibrary.com]

Third, scalp maps of the input‐perturbation effects on network predictions for the different frequency bands, as shown in Figure 19, show spatial distributions expected for motor tasks in the alpha, beta and—for the first time for such a noninvasive EEG decoding visualization—for the high gamma band. These scalp maps directly reflect the behavior of the ConvNets and one needs to be careful when making inferences about the data from them. For example, the positive correlation on the right side of the scalp for the Hand (R) class in the alpha band only means the ConvNet increased its prediction when the amplitude at these electrodes was increased independently of other frequency bands and electrodes. It does not imply that there was an increase of amplitude for the right hand class in the data. Rather, this correlation could be explained by the ConvNet reducing common noise between both locations, for more explanations of these effects in case of linear models, see Haufe et al. [2014]. Nevertheless, for the first time in noninvasive EEG, these maps clearly revealed the global somatotopic organization of causal contributions of motor cortical gamma band activity to decoding right and left hand and foot movements. Interestingly, these maps revealed highly focalized patterns, particularly during hand movement in the gamma frequency range (Fig. 16, first plots in last row), in contrast to the more diffuse patterns in the conventional task‐related spectral analysis as shown in Figure 14.

In summary, our visualization methods proved useful to map the spatial distribution of the features learned by the ConvNets to perform single‐trial decoding of the different movement classes and in different physiologically important frequency bands.

## DISCUSSION

This study systematically evaluated ConvNet of different architectures and with different design choices against a validated baseline method, that is, FBCSP. This study shows that ConvNets allow accurate task decoding from EEG, that recent deep‐learning techniques are critical to boost ConvNet performance, and that a cropped ConvNet training strategy can further increase decoding performance. Thus, ConvNets can achieve successful end‐to‐end learning from EEG with just minimal preprocessing. This study also demonstrates that novel ConvNets visualization offer new possibilities in brain mapping of informative EEG features.

### Architectures and Design Choices

#### ConvNets versus FBCSP

Our results demonstrate that deep and shallow ConvNets, with appropriate design choices, are able to—at least—reach the accuracies of FBCSP for motor decoding from EEG (see Result 2). In our main comparison for the combined datasets (Table 2), the accuracies of both deep and shallow ConvNets are very close and slightly higher than the accuracies of FBCSP. As filter bank common spatial patterns is the de facto standard for motor decoding from EEG recordings, this strongly implies ConvNets are also a suitable method for motor decoding. While we have shown deep ConvNets to be competitive with standard FBCSP, a lot of variants of FBCSP exist. For example, many regularized variants of CSP exist that can be used inside FBCSP [Lotte and Guan, 2011; Samek, 2014]; a comparison to these could further show the exact tradeoff between the more generic ConvNets and the more domain‐specific FBCSP.

#### Role of recent deep learning advances

Success depends on using recent developments in deep learning. The accuracy increase that we demonstrate when using batch normalization, dropout and exponential linear units implies that general advances in deep learning can also improve brain‐signal decoding. The improvement from using these techniques replicates recent findings in computer vision and other fields. In our study, improvements were most pronounced for the deep ConvNet on 4–*f_end_*Hz data (Result 6), indicating that the networks can easily overfit in this setting, where band power features are likely dominant. This is consistent with our observation that cropped training, which combats overfitting by increasing the number of training examples, also drastically increased accuracies on 4–*f_end_*Hz data (Result 8). There seemed to be some further gains when combining both batch normalization and dropout, albeit with some variation across architectures and frequency bands. This improvement was not clear from the start as batch normalization can in some cases remove the need for dropout [Ioffe and Szegedy, 2015], however this improvement was also found in another study using ConvNets to decode EEG data [Lawhern et al., 2016]. The smaller improvement batch normalization yielded for the deep ConvNet is consistent with the claim that ELUs already allow fast learning [Clevert et al., 2016]. However, all these findings are limited by the fact that there can be interactions between these methods and with all other hyperparameters. As of yet, we also do not have a clear explanation for the large difference in accuracies obtained with ReLUs compared to ELUs; a recent study on computer vision tasks did not find these differences [Mishkin et al., 2016]. Mathematically and empirically analyzing the behavior of ELUs and ReLUs for oscillatory signals and typical EEG noise might shed some light on plausible reasons.

#### ConvNet architectures and interactions with discriminative features

Another finding of our study was that the shallow ConvNets performed as good as the deep ConvNets, in contrast to the hybrid and residual architectures (see Results 2, 4, and 7). These observations could possibly be better understood by investigating more closely what discriminative features there are in the EEG data and what architectures can hence best use them. For example, it would be interesting to study the effect of more layers when the networks use mostly EEG band power features, phase‐related features, or a combination thereof (c.f. Hammer et al. [2013], for the role of power and phase in motor decoding) and whether there are features for which a deeper hierarchical representation could be beneficial.

We observed that squaring was important for the shallow but not for the deep ConvNet (Result 5). The worse performance of the shallow ConvNet with ELU instead of squaring may be explained as follows. Squaring naturally allows the network to more easily extract band power features: In combination with the approximately zero‐mean input, the network would already capture the signal's variance by squaring. To see this, assume that the two bandpass‐filter‐like and spatial‐filter‐like convolutional layers extract an oscillatory source in a specific frequency band; the squaring and mean pooling then directly computes the variance of this source in the pool regions. With ELUs instead of squaring, the positive parts of the oscillation would remain unchanged while the negative ones would be suppressed; the mean of the pool region would still be larger for larger amplitudes of the oscillation, but less strongly so than for the square activation. The effects of ELU and squaring for the deep ConvNet are less straightforward to analyze, as the pooling regions in our deep ConvNet were much smaller than for the shallow ConvNet (3 vs 75 samples) and might thus not cover a large enough time span to compute a very robust and informative variance average.

#### Possibilities for substantial decoding accuracy improvements

In the analyses presented here, ConvNets did not improve accuracies over FBCSP by a large margin. Significant improvements, if present, were never larger than 3.5% on the combined dataset with a lot of variation per subject (Result 2). However, the deep ConvNets as used here may have learned features different from FBCSP, which could explain their higher accuracies in the lower frequencies where band power features may be less important [Hammer et al., 2013]. Nevertheless, ConvNets failed to clearly outperform FBCSP in our experiments. Several reasons might contribute to this: the datasets might still not be large enough to reveal the full potential of deeper convolutional networks in EEG decoding; or the class‐discriminative features might not have enough hierarchical structure which deeper ConvNets could exploit. The dataset‐size issue could be solved by either creating larger datasets or also by using transfer learning approaches across subjects and/or other datasets. Further analysis of the data itself and of the convolutional networks might help to shed light whether there are features with a lot of hierarchical structure. Finally, recurrent networks could exploit signal changes that happen on longer timescales, for example, electrodes slowly losing scalp contact over the course of a session, changes of the electrode cap position or nonstationarities in the brain signals. Thus, there is clearly still a large potential for methodological improvement in ConvNet‐based EEG decoding.

These methodological improvements might also come from further methodological advances in deep learning, such as newer forms of hyperparameter optimization, in case these advances also translate to even better EEG decoding accuracies. As discussed above, recent advances like dropout, batch normalization and exponential linear units can substantially improve the performance of EEG decoding with ConvNets, especially for our deep architecture. Therefore, using other recent techniques, such as newer forms of hyperparameter optimization [Domhan et al., 2015; Klein et al., 2016; Springenberg et al., 2016] hold promise to further increase accuracies of ConvNets for brain‐signal decoding. Furthermore, as the field is still evolving at a fast pace, new techniques can be expected to be developed and might then also benefit brain‐signal decoders using convolutional neural networks.

However, methodological improvements may also happen in the broad field of “non‐ConvNet” approaches. Obviously, currently no final verdict is possible about an “optimal” method for EEG decoding if there is a single best method for the large variety of EEG decoding problems at all. The findings of this study, however, support that ConvNet‐based decoding is a contender in this competition.

#### Further potential advantages of ConvNets for brain‐signal decoding

Besides the decoding performance, there are also other potential advantages of using deep ConvNets for brain‐signal decoding. First, several use cases desirable for brain‐signal decoding are very easy to do with deep ConvNets iteratively trained in an end‐to‐end fashion: Deep ConvNets can be applied to other types of tasks such as as workload estimation, error‐ or event‐related potential decoding (as others have started [Lawhern et al., 2016]) or even other types of recordings such as MEG or ECoG. Also, ConvNets, due to their iterative training, have a natural way of pretraining and finetuning; for example, a ConvNet can be pretrained on data from the past or data from other subjects and then be finetuned with new data from a new subject. Finetuning can be as simple as continuing the iterative training process on the new data, possibly with a smaller learning rate and this finetuning can also be used to perform supervised online adaptation. Second, due to their joint optimization, single ConvNets can be building blocks for more sophisticated setups of multiple ConvNets. One recent example attempts to create ConvNets that are robust to changes in the input distribution [Ganin et al., 2016]. This could be used to alleviate the long‐standing EEG decoding problem of changes in the EEG signal distribution from one session to another.

#### Limitations of ConvNets for brain‐signal decoding

The flexibility of ConvNets might also be a limitation in some brain‐signal decoding scenarios. For example if the user of a brain–computer interface should learn to adapt her or his brain signals to the decoding model, a simpler feature‐based model might yield better results. One example of this would be BCI rehabilitation where the user should learn to reinforce a certain brain activity pattern [Daly and Wolpaw, 2008]. A similar example would be motor decoding brain‐computer interface that are using single cell activity, where researchers have argued a linear model is the best decoding model [Collinger et al., 2013; Georgopoulos et al., 1986; Hochberg et al., 2012]. Studies comparing ConvNets with other techniques in these areas would be an interesting and practically relevant line of further research.

### Training Strategy

#### Cropped training effect on accuracies

We observed that cropped training was necessary for the deep ConvNet to reach competitive accuracies on the dataset excluding very low frequencies (Result 8). The large increase in accuracy with cropped training for the deep network on the 4–*f_end_*‐Hz data might indicate a large number of training examples is necessary to learn to extract band power features. This makes sense as the shifted neighboring windows may contain the same, but shifted, oscillatory signals. These shifts could prevent the network from overfitting on phase information within the trial, which is less important in the higher than the lower frequencies [Hammer et al., 2013]. This could also explain why other studies on ConvNets for brain‐signal decoding, which did not use cropped training, but where band power might be the most discriminative feature, have used fairly shallow architectures and sometimes found them to be superior to deeper versions [Stober et al., 2014].

#### Suitability for online decoding

Our cropped training strategy appears particularly well‐applicable for online brain‐signal decoding. As described above, it may offer performance advantages compared with conventional (noncropped) training. Additionally, cropped training allows for a useful calibration of the tradeoff between decoding delay and decoding accuracy in online settings. The duration from trial start until the last sample of the first crop should roughly correspond to the minimum time needed to decode a control signal. Hence, smaller crops can allow less delay—the first small crop could end at an early sample within the trial without containing too many timesteps from before the trial that could otherwise disturb the training process. Conversely, larger crops that still contain mostly timesteps from within the trial imply a larger delay until a control signal is decoded while possibly increasing the decoding accuracy due to more information contained in the larger crops. These intuitions should be confirmed in online experiments.

### Visualization

#### Insights from current visualizations

In addition to exploring how ConvNets can be successfully used to decode information from the EEG, we have also developed and tested two complementary methods to visualize what ConvNets learn from the EEG data. So far, the literature on using ConvNets for brain‐signal decoding has, for example, visualized weights or outputs of ConvNet layers [Bashivan et al., 2016; Santana et al., 2014; Stober, 2016; Yang et al., 2015], determined inputs that maximally activate specific convolutional filters [Bashivan et al., 2016], or described attempts at synthesizing the preferred input of a convolutional filter [Bashivan et al., 2016] (see Supporting Information, Section A.1 for a more extensive overview). Here, we applied both a correlative and a causally interpretable visualization method to visualize the frequencies and spatial distribution of band power features used by the networks.

The visualizations showed plausible, spatially localized spatial distributions for motor tasks in the alpha, beta and gamma bands (see the section “Visualization”). The input‐feature unit‐output and the input‐perturbation network‐prediction correlation maps together clearly showed that the deep ConvNet learned to extract and use band power features with specific, physiologically plausible spatial distributions. This also indicates that the ConvNets were using brain signals to decode the EEG signal and were not primarily relying on artifactual components. Hence, while the computation of power was built into both the FBCSP and shallow ConvNet, our deep ConvNets successfully learned to perform the computation of band power features from the raw input in an end‐to‐end manner. Our network correlation maps can readily show spatial distributions per subject and for the whole group of subjects. Interestingly, the input‐perturbation network‐prediction correlation maps for the deep ConvNets revealed highly focalized patterns, particularly during hand movement in the gamma frequency range (Fig. 19, first plots in last row). This contrasted to the more diffuse patterns in the conventional task‐related spectral analysis as shown in Figure 14 and suggests that ConvNet visualization may be useful for task‐related brain mapping in the spectral domain, possibly with improved localization power as compared to traditional techniques for mapping task‐related spectral EEG modulations.

#### Feature discovery through more sophisticated visualizations

We designed the visualizations presented here to show how ConvNets use the amplitude of spectral band power features. One straightforward extension would be to apply these visualizations to show how ConvNets use the amplitude of the raw time‐domain EEG signal. This could give insights into discriminative time‐domain features such as event‐related potentials. A slightly more involved extension would be to apply them on circular features such as phase features. Moreover, it could be even more interesting to investigate whether novel or so‐far unknown features are used and to characterize them. This could be especially informative for tasks where the discriminative features are less well known than for motor decoding, for example, for less‐investigated tasks such as decoding of task performance [Meinel et al., 2016]. But even for the data used in this study, our results show hints that deep ConvNets used different features than shallow ConvNets and the FBCSP‐based decoding, as there are statistically significant differences between their confusion matrices (Result 3). This further strengthens the motivation to explore what features the deep ConvNet exploits, for example, using visualizations that show what parts of a trial are relevant for the classification decision or what a specific convolutional filter/unit output encodes. Newer visualization methods such as layer‐wise relevance propagation [Bach et al., 2015; Montavon et al., 2017], inverting convolutional networks with convolutional networks [Dosovitskiy and Brox, 2016] or synthesizing preferred inputs of units [Nguyen et al., 2016] could be promising next steps in that direction.

### Conclusion

In conclusion, ConvNets are not only a novel, promising tool in the EEG decoding toolbox, but combined with innovative visualization techniques, they may also open up new windows for EEG‐based brain mapping.

## CONFLICTS OF INTEREST

The authors declare that there is no conflict of interest regarding the publication of this article.

## Supporting information

Supporting Information
