# Supplementary material for: Deep learning with convolutional neural networks for EEG decoding and visualization
Source: Hum Brain Mapp. 2017 Aug 7;38(11):5391–420. doi: 10.1002/hbm.23730 (PMC5655781; doi:10.1002/hbm.23730)
Supplement: Supplementary file 1 — Supporting Information [file HBM-38-5391-s001.pdf]

Supplementary for: Deep learning with convolutional neural networks for EEG decoding and visualization

Short title: *Convolutional neural networks in EEG analysis*

Keywords: *Electroencephalography, EEG analysis, machine learning, end-to-end learning, brain-machine interface (BCI), brain-computer interface (BMI), model interpretability, brain mapping*

Robin Tibor Schirrmeister[1 a,b], Jost Tobias Springenberg[c,b], Lukas Dominique Josef Fiederer[a,b,d], Martin Glasstetter[a,b], Katharina Eggensperger[e,b], Michael Tangermann[f,b], Frank Hutter[e,b], Wolfram Burgard[g,b], and Tonio Ball[a,b]

[a] *Translational Neurotechnology Lab, Epilepsy Center, Medical Center – University of Freiburg, Engelberger Str. 21, 79106 Freiburg, Germany*
[b] *BrainLinks-BrainTools Cluster of Excellence, University of Freiburg, Georges-Köhler-Allee 79, 79110 Freiburg, Germany*
[c] *Machine Learning Lab, Computer Science Dept., University of Freiburg, Georges-Köhler-Allee 79, 79110 Freiburg, Germany*
[d] *Neurobiology and Biophysics, Faculty of Biology, University of Freiburg, Hansastr. 9a, 79104 Freiburg, Germany*
[e] *Machine Learning for Automated Algorithm Design Lab, Computer Science Dept., University of Freiburg, Georges-Köhler-Allee 52, 79110 Freiburg im Breisgau, Germany*
[f] *Brain State Decoding Lab, Computer Science Dept., University of Freiburg, Albertstr. 23, 79104 Freiburg, Germany*
[g] *Autonomous Intelligent Systems Lab, Computer Science Dept., University of Freiburg, Georges-Köhler-Allee 79, 79110 Freiburg, Germany*

August 7, 2017

[1] Corresponding author: robin.schirrmeister@uniklinik-freiburg.de

A Supplementary Materials

A.1 Related Work

Study	Decoding problem	Input domain	Conv/ dense layers	Design choices	Training strategies	External baseline	Visualization type(s)	Visualization findings
This manuscript, Schirrmeister et. al (2017)	Imagined and executed movement classes, within subject	Time, 0–125 Hz	5/1	Different ConvNet architectures<br>Nonlinearities and pooling modes<br>Regularization and intermediate normalization layers<br>Factorized convolutions<br>Splitted vs one-step convolutions	Trial-wise vs. cropped training strategy	FBCSP + rLDA	Feature activation correlation<br>Feature-perturbation prediction correlation	See Section 3.5
Single-trial EEG classification of motor imagery using deep convolutional neural networks, Tang et al. (2017)	Imagined movement classes, within-subject	Time, 8–30 Hz	2/2			FBCSP		
EEGNet: A Compact Convolutional Network for EEG-based Brain-Computer Interfaces, Lawhern et al. (2016)	Oddball response (RSVP), error response (ERN), movement classes (voluntarily started and imagined)	Time, 0.1–40 Hz	3/1	Kernel sizes				
Remembered or Forgotten? — An EEG-Based Computational Prediction Approach, Sun et al. (2016)	Memory performance, within-subject	Time, 0.05–15 Hz	2/2		Different time windows		Weights (spatial)	Largest weights found over prefrontal and temporal cortex
Multimodal Neural Network for Rapid Serial Visual Presentation Brain Computer Interface, Manor et al. (2016)	Oddball response using RSVP and image (combined image-EEG decoding), within-subject	Time, 0.3–20 Hz	3/2				Weights<br>Activations<br>Saliency maps by gradient	Weights showed typical P300 distribution<br>Activations were high at plausible times (300-500ms)<br>Saliency maps showed plausible spatio-temporal plots
A novel deep learning approach for classification of EEG motor imagery signals, Tabar and Halici (2017)	Imagined and executed movement classes, within-subject	Frequency, 6–30 Hz	1/1	Addition of six-layer stacked autoencoder on ConvNet features<br>Kernel sizes		FBCSP, Twin SVM, DDFBS, Bispectrum, RQNN	Weights (spatial + frequential)	Some weights represented difference of values of two electrodes on different sides of head

Study	Decoding problem	Input domain	Conv/ dense layers	Design choices	Training strategies	External baseline	Visualization type(s)	Visualization findings
Predicting Seizures from Electroencephalography Recordings: A Knowledge Transfer Strategy, Liang et al. (2016)	Seizure prediction, within-subject	Frequency, 0–200 Hz	1/2		Different subdivisions of frequency range<br>Different lengths of time crops<br>Transfer learning with auxiliary non-epilepsy datasets		Weights<br>Clustering of weights	Clusters of weights showed typical frequency band subdivision (delta, theta, alpha, beta, gamma)
EEG-based prediction of driver's cognitive performance by deep convolutional neural network, Hajinoroozi et al. (2016)	Driver performance, within- and cross-subject	Time, 1–50 Hz	1/3	Replacement of convolutional layers by restricted Boltzmann machines with slightly varied network architecture				
Deep learning for epileptic intracranial EEG data, Antoniades et al. (2016)	Epileptic discharges, cross-subject	Time, 0–100 HZ	1–2/2	1 or 2 convolutional layers			Weights<br>Correlation weights and interictal epileptic discharges (IED)<br>Activations	Weights increasingly correlated with IED waveforms with increasing number of training iterations<br>Second layer captured more complex and well-defined epileptic shapes than first layer<br>IEDs led to highly synchronized activations for neighbouring electrodes
Learning Robust Features using Deep Learning for Automatic Seizure Detection, Thodoroff et al. (2016)	Start of epileptic seizure, within- and cross-subject	Frequency, mean amplitude for 0–7 Hz, 7–14 Hz, 14–49 Hz	3/1 (+ LSTM as postprocessor)			Hand crafted features + SVM	Input occlusion and effect on prediction accuracy	Allowed to locate areas critical for seizure
Single-trial EEG RSVP classification using convolutional neural networks, Shamwell et al. (2016)	Oddball response (RSVP), groupwise (ConvNet trained on all subjects)	Time, 0.5–50 Hz	4/3				Weights (spatial)	Some filter weights had expected topographic distributions for P300<br>Others filters had large weights on areas not traditionally associated with P300
Wearable seizure detection using convolutional neural networks with transfer learning, Page et al. (2016)	Seizure detection, cross-subject, within-subject, groupwise	Time, 0–128 Hz	1-3/1-3		Cross-subject supervised training, within-subject finetuning of fully connected layers	Multiple: spectral features, higher order statistics + linear-SVM, RBF-SVM, ...		

Study	Decoding problem	Input domain	Conv/dense layers	Design choices	Training strategies	External baseline	Visualization type(s)	Visualization findings
Learning Representations from EEG with Deep Recurrent-Convolutional Neural Networks, Bashivan et al. (2016)	Cognitive load (number of characters to memorize), cross-subject	Frequency, mean power for 4–7 Hz, 8–13 Hz, 13–30 Hz	3–7/2 (+ LSTM or other temporal post-processing (see design choices))	Number of convolutional layers<br>Temporal processing of ConvNet output by max pooling, temporal convolution, LSTM or temporal convolution + LSTM			Inputs that maximally activate given filter<br>Activations of these inputs<br>"Deconvolution" for these inputs	Different filters were sensitive to different frequency bands<br>Later layers had more spatially localized activations<br>Learned features had noticeable links to well-known electrophysiological markers of cognitive load
Deep Feature Learning for EEG Recordings, Stober (2016)	Type of music rhythm, groupwise (ensembles of leave-one-subject-out trained models, evaluated on separate test set of same subjects)	Time, 0.5–30Hz	2/1	Kernel sizes	Pretraining first layer as convolutional autoencoder with different constraints		Weights (spatial+3 timesteps, pretrained as autoencoder)	Different constraints led to different weights, one type of constraints could enforce weights that are similar across subjects; other type of constraints led to weights that have similar spatial topographies under different architectural configurations and preprocessings
Convolutional Neural Network for Multi-Category Rapid Serial Visual Presentation BCI, Manor and Geva (2015)	Oddball response (RSVP), within-subject	Time, 0.1–50 Hz	3/3 (Spatio-temporal regularization)				Weights<br>Mean and single-trial activations	Spatiotemporal regularization led to softer peaks in weights<br>Spatial weights showed typical P300 distribution<br>Activations mostly had peaks at typical times (300-400ms)
Parallel Convolutional-Linear Neural Network for Motor Imagery Classification, Sakhavi et al. (2015)	Imagined movement classes, within-subject	Frequency, 4–40 Hz, using FBCSP	2/2 (Final fully connected layer uses concatenated output by convolutional and fully connected layers)	Combination ConvNet and MLP (trained on different features) vs. only ConvNet vs. only MLP				
Using Convolutional Neural networks to Recognize Rhythm Stimuli form Electroencephalography Recordings, Stober et al. (2014)	Type of music rhythm, within-subject	Time and frequency evaluated, 0-200 Hz	1-2/1	Best values from automatic hyperparameter optimization: frequency cutoff, one vs two layers, kernel sizes, number of channels, pooling width	Best values from automatic hyperparameter optimization: learning rate, learning rate decay, momentum, final momentum			

Study	Decoding problem	Input domain	Conv/ dense layers	Design choices	Training strategies	External baseline	Visualization type(s)	Visualization findings
Convolutional deep belief networks for feature extraction of EEG signal, Ren and Wu (2014)	Imagined movement classes, within-subject	Frequency, 8–30 Hz	2/0 (Convolutional deep belief network, separately trained RBF-SVM classifier)					
Deep feature learning using target priors with applications in ECoG signal decoding for BCI, Wang et al. (2013)	Finger flexion trajectory (regression), within-subject	Time, 0.15–200 Hz	3/1 (Convolutional layers trained as convolutional stacked autoencoder with target prior)	Partially supervised CSA				
Convolutional neural networks for P300 detection with application to brain-computer interfaces, Cecotti and Graser (2011)	Oddball / attention response using P300 speller, within-subject	Time, 0.1-20 Hz	2/2	Electrode subset (fixed or automatically determined)<br>Using only one spatial filter<br>Different ensembling strategies		Multiple: Linear SVM, gradient boosting, E-SVM, S-SVM, mLVQ, LDA, ...	Weights	Spatial filters were similar for different architectures<br>Spatial filters were different (more focal, more diffuse) for different subjects

Table S1: **Related previous publications using convolutional neural networks for EEG decoding.** Frequency domain input always only contains amplitudes or a transformation of amplitudes (power, log power, etc.), never phase information. The number of dense layers includes parametrized classification layers. Layer numbers always refer to EEG decoding models in cases of articles that use multiple modalities for decoding. Special features of the model written in parentheses after the number of layers, especially if these features make the number of layers misleading. External baseline: the study includes directly comparable baseline accuracies of non-deep-learning approaches of other authors. Visualization types and findings both refer to visualizations of the trained networks used for EEG decoding; findings are paraphrased from the original publications. Note that none of the previous studies using time-domain input showed, using a suitable visualization technique, that the ConvNets learned to use band power features, in contrast to our present study. Also note that other previous studies used artificial neural networks without convolutions for EEG analysis, e.g. Santana et al. (2014); Sturm et al. (2016).

A.2 FBCSP implementation

As in many previous studies (Lotte et al., 2007), we used regularized linear discriminant analysis (RLDA) as the classifier, with shrinkage regularization (Ledoit and Wolf, 2004). To decode multiple classes, we used one-vs-one majority weighted voting: We trained an RLDA classifier for each pair of classes, summed the classifier outputs (scaled to be in the same range) across classes and picked the class with the highest sum (Chin et al., 2009; Galar et al., 2011).

FBCSP is typically used with feature selection, since few spatial filters from few frequency bands often suffice to reach good accuracies and using many or even all spatial filters often leads to overfitting (Blankertz et al., 2008; Chin et al., 2009). We use a classical measure for preselecting spatial filters, the ratio of the corresponding power features for both classes extracted by each spatial filter (Blankertz et al., 2008). Additionally, we performed a feature selection step on the final filter bank features by selecting features using an inner cross validation on the training set, see published code [1] for details.

In the present study, we designed two filter banks adapted for the two datasets to capture most discriminative motor-related band power information. In preliminary experiments on the training set, overlapping frequency bands led to higher accuracies, as also proposed by Sun et al. (2010). As the bandwidth of physiological EEG power modulations typically increases in higher frequency ranges (Buzsáki and Draguhn, 2004), we used frequency bands with 6 Hz width and 3 Hz overlap in frequencies up to 13 Hz, and bands of 8 Hz width and 4 Hz overlap in the range above 10 Hz. Frequencies above 38 Hz only improved accuracies on one of our datasets, the so-called High-Gamma Dataset (see Section 2.7, where we also describe the likely reason for this difference, namely that the recording procedure for the High-Gamma Dataset — but not for the BCI competition datasets — was specifically optimized for the high frequency range). Hence the upper limit of used frequencies was set at 38 Hz for the BCI competition datasets, while the upper limit for the High-Gamma Dataset was set to 122 Hz, close to the Nyquist frequency, thus allowing FBCSP to also use information from the gamma band.

As a sanity check, we compared the accuracies of our FBCSP implementation to those published in the literature for the same BCI competition IV dataset 2a (Sakhavi et al., 2015), showing very similar

[1] https://github.com/robintibor/braindecode/blob/f9497f96a6dfdea1e24a4709a9ceb30e0f4768e3/braindecode/csp/pipeline.py#L200-L408

performance: 67.59% for our implementation vs 67.01% for their implementation on average across subjects (p>0.7, Wilcoxon signed-rank test, see Result 1 for more detailed results). This underlines that our FBCSP implementation, including our feature selection and filter bank design, indeed was a suitable baseline for the evaluation of our ConvNet decoding accuracies.

A.3 Residual network architecture

In total, the ResNet has 31 convolutional layers, a depth where ConvNets without residual blocks started to show problems converging in the original ResNet paper (He et al., 2015). In layers where the number of channels is increased, we padded the incoming feature map with zeros to match the new channel dimensionality for the shortcut, as in option A of the original paper (He et al., 2015).

Layer/Block	Number of Kernels	Kernel Size	Output Size
Input			1000x44x1
Convolution (linear)	48	3x1	1000x44x48
Convolution (ELU)	48	1x44	1000x1x48
ResBlock (ELU)	48	3x1	
ResBlock (ELU)	48	3x1	
ResBlock (ELU)	96	3x1 (Stride 2x1)	500x1x96
ResBlock (ELU)	96	3x1	
ResBlock (ELU)	144	3x1 (Stride 2x1)	250x1x96
ResBlock (ELU)	144	3x1	
ResBlock (ELU)	144	3x1 (Stride 2x1)	125x1x96
ResBlock (ELU)	144	3x1	
ResBlock (ELU)	144	3x1 (Stride 2x1)	63x1x96
ResBlock (ELU)	144	3x1	
ResBlock (ELU)	144	3x1 (Stride 2x1)	32x1x96
ResBlock (ELU)	144	3x1	
ResBlock (ELU)	144	3x1 (Stride 2x1)	16x1x96
ResBlock (ELU)	144	3x1	
Mean Pooling		10x1	7x1x144
Convolution + Softmax	4	1x1	7x1x4

Table S2: **Residual network architecture hyperparameters**. Number of kernels, kernel and output size for all subparts of the network. Output size is always time x height x channels. Note that channels here refers to input channels of a network layer, not to EEG channels; EEG channels are in the height dimension. Output size is only shown if it changes from the previous block. Second convolution and all residual blocks used ELU nonlinearities. Note that in the end we had seven outputs, i.e., predictions for the four classes, in the time dimension (**7**x1x4 final output size). In practice, when using cropped training as explained in Section 2.5.4, we even had 424 predictions, and used the mean of these to predict the trial.

A.4 Optimization and early stopping

Adam is a variant of stochastic gradient descent designed to work well with high-dimensional parameters, which makes it suitable for optimizing the large number of parameters of a ConvNet (Kingma and Ba, 2014). The early stopping strategy that we use throughout this study, developed in the computer vision field [2], splits the training set into a training and validation fold and stops the first phase of the training when validation accuracy does not improve for a predefined number of epochs. The training continues on the combined training and validation fold starting from the parameter values that led to the best accuracies on the validation fold so far. The training ends when the loss function on the validation fold drops to the same value as the loss function on the training fold at the end of the first training phase (we do not continue training in a third phase as in the original description). Early stopping in general allows training on different types of networks and datasets without choosing the number of training epochs by hand. Our specific strategy uses the entire training data while only training once. In our study, all reported accuracies have been determined on an independent test set.

A.5 Visualization methods

A.5.1 EEG spectral power topographies

To visualize the class-specific EEG spectral power modulations, we computed band-specific envelope-class correlations in the alpha, beta and gamma bands for all classes of the High-Gamma Dataset. The group-averaged topographies of these maps could be readily compared to our input-feature unit-output network correlation maps, since, similar to the power-class correlation map described in Section 2.6.2, we computed correlations of the moving average of the squared envelope with the actual class labels, using the receptive field size of the final layer as the moving average window size. Since this is a ConvNet-independent visualization, we did not subtract any values of an untrained ConvNet. We show the resulting scalp maps for the four classes and did not average over them. Note that these computations were only used for the power topographies shown in Figure 14 and did not enter the decoding analyses as described in the preceding sections.

[2] https://web.archive.org/web/20160809230156/https://code.google.com/p/cuda-convnet/wiki/Methodology

A.6 Dataset details

The BCI competition IV dataset 2a is a 22-electrode EEG motor-imagery dataset, with 9 subjects and 2 sessions, each with 288 four-second trials of imagined movements per subject (movements of the left hand, the right hand, the feet and the tongue) (Brunner et al., 2008). The training set consists of the 288 trials of the first session, the test set of the 288 trials of the second session.

The BCI competition IV dataset 2b is a 3-electrode EEG motor-imagery dataset with 9 subjects and 5 sessions of imagined movements of the left or the right hand, the latest 3 sessions include online feedback (Leeb et al., 2008). The training set consists of the approx. 400 trials of the first 3 sessions (408.9±13.7, mean±std), the test set consists of the approx. 320 trials (315.6±12.6, mean±std) of the last two sessions.

Our "High-Gamma Dataset" is a 128-electrode dataset (of which we later only use 44 sensors covering the motor cortex, (see Section 2.7.1), obtained from 14 healthy subjects (6 female, 2 left-handed, age 27.2±3.6 (mean±std)) with roughly 1000 (963.1±150.9, mean±std) four-second trials of executed movements divided into 13 runs per subject. The four classes of movements were movements of either the left hand, the right hand, both feet, and rest (no movement, but same type of visual cue as for the other classes). The training set consists of the approx. 880 trials of all runs except the last two runs, the test set of the approx. 160 trials of the last 2 runs. This dataset was acquired in an EEG lab optimized for non-invasive detection of high-frequency movement-related EEG components (Ball et al., 2008; Darvas et al., 2010). Such high-frequency components in the range of approx. 60 to above 100 Hz are typically increased during movement execution and may contain useful movement-related information (Crone et al., 1998; Hammer et al., 2016; Quandt et al., 2012). Our technical EEG Setup comprised (1.) Active electromagnetic shielding: optimized for frequencies from DC - 10 kHz (-30 dB to -50 dB), shielded window, ventilation & cable feedthrough (mrShield, CFW EMV-Consulting AG, Reute, CH) (2.) Suitable amplifiers: high-resolution (24 bits/sample) and low-noise (<0.6 μV RMS 0.16–200 Hz, <1.5 μV RMS 0.16–3500 Hz), 5 kHz sampling rate (NeurOne, Mega Electronics Ltd, Kuopio, FI) (3.) actively shielded EEG caps: 128 channels (WaveGuard Original, ANT, Enschede, NL) and (4.) full optical decoupling: All devices are battery powered and communicate via optic fibers.

Subjects sat in a comfortable armchair in the dimly lit Faraday cabin. The contact impedance from electrodes to skin was typically reduced below 5 kOhm using electrolyte gel (SUPER-VISC, EASYCAP

GmbH, Herrsching, GER) and blunt cannulas. Visual cues were presented using a monitor outside the cabin, visible through the shielded window. The distance between the display and the subjects' eyes was approx. 1 m. A fixation point was attached at the center of the screen. The subjects were instructed to relax, fixate the fixation mark and to keep as still as possible during the motor execution task. Blinking and swallowing was restricted to the inter-trial intervals. The electromagnetic shielding combined with the comfortable armchair, dimly lit Faraday cabin and the relatively long 3-4 second inter-trial intervals (see below) were used to minimize artifacts produced by the subjects during the trials.

The tasks were as following. Depending on the direction of a gray arrow that was shown on black background, the subjects had to repetitively clench their toes (downward arrow), perform sequential finger-tapping of their left (leftward arrow) or right (rightward arrow) hand, or relax (upward arrow). The movements were selected to require little proximal muscular activity while still being complex enough to keep subjects involved. Within the 4-s trials, the subjects performed the repetitive movements at their own pace, which had to be maintained as long as the arrow was showing. Per run, 80 arrows were displayed for 4 s each, with 3 to 4 s of continuous random inter-trial interval. The order of presentation was pseudo-randomized, with all four arrows being shown every four trials. Ideally 13 runs were performed to collect 260 trials of each movement and rest. The stimuli were presented and the data recorded with BCI2000 (Schalk et al., 2004). The experiment was approved by the ethical committee of the University of Freiburg.

The Mixed Imagery Dataset (MID) was obtained from 4 healthy subjects (3 female, all right-handed, age 26.75±5.9 (mean±std)) with a varying number of trials (S1: 675, S2: 2172, S3: 698, S4: 464) of imagined movements (right hand and feet), mental rotation and mental word generation. All details were the same as for the High Gamma Dataset, except: a 64-electrode subset of electrodes was used for recording, recordings were not performed in the electromagnetically shielded cabin, thus possibly better approximating conditions of real-world BCI usage, and trials varied in duration between 1 to 7 seconds. The dataset was analyzed by cutting out time windows of 2 seconds with 1.5 second overlap from all trials longer than 2 seconds (S1: 6074 windows, S2: 21339, S3: 6197, S4: 4220), and both methods were evaluated using the accuracy of the predictions for all the 2-second windows for the last two runs of roughly 130 trials (S1: 129, S2: 160, S3: 124, S4: 123).

A.7 EEG preprocessing

We resampled the High-Gamma Dataset to 250 Hz, i.e., the same as the BCI competition datasets, to be able to use the same ConvNet hyperparameter settings for both datasets. To ensure that the ConvNets only have access to the same frequency range as the CSPs, we low-pass filtered the BCI competition datasets to below 38 Hz. In case of the 4–f_{end}-Hz dataset, we highpass-filtered the signal as described in 2.7.1 (for the BCI competition datasets, we *bandpass-filtered* to 4-38 Hz, so the previous lowpass-filter step was merged with the highpass-filter step). Afterwards, for both sets, for the ConvNets, we performed electrode-wise exponential moving standardization with a decay factor of 0.999 to compute exponential moving means and variances for each channel and used these to standardize the continuous data. Formally,

$$x't = (x_t - \mu_t)/\sqrt{\sigma_t^2} \tag{1}$$

$$\mu_t = 0.001x_t + 0.999\mu_{t-1} \tag{2}$$

$$\sigma_t^2 = 0.001(x_t - \mu_t)^2 + 0.999\sigma_{t-1}^2 \tag{3}$$

where $x't$ and x_t are the standardized and the original signal for one electrode at time t, respectively. As starting values for these recursive formulas we set the first 1000 mean values μ_t and first 1000 variance values σ_t^2 to the mean and the variance of the first 1000 samples, which were always completely inside the training set (so we never used future test data in our preprocessing). Some form of standardization is a commonly used procedure for ConvNets; exponentially moving standardization has the advantage that it is also applicable for an online BCI. For FBCSP, this standardization always worsened accuracies in preliminary experiments, so we did not use it. We also did not use the standardization for our visualizations to ensure that the standardization does not make our visualizations harder to interpret. Overall, the minimal preprocessing without any manual feature extraction ensured our end-to-end pipeline could in principle be applied to a large number of brain-signal decoding tasks.

We also only minimally cleaned the datasets to remove extreme high-amplitude recording artifacts. Our cleaning method thus only removed trials where at least one channel had a value outside $\pm 800\ \mu V$. We kept trials with lower-amplitude artifacts as we assumed these trials might still contain useful brain-signal information. As described in Sections 2.6 and 3.5, we used visualization of the features learned by the

ConvNets to verify that they learned to classify brain signals and not artifacts. Furthermore, for the High-Gamma Dataset, we used only those sensors covering the motor cortex: all central electrodes (45), except the Cz electrode which served as the recording reference electrode. Interestingly, using all electrodes led to worse accuracies for both the ConvNets and FBCSP, which may be a useful insight for the design of future movement-related decoding/BCI studies. Any further data restriction (trial-or channel-based cleaning) never led to accuracy increases in either of the two methods when averaged across all subjects. For the visualizations, we used all electrodes and common average re-referencing to investigate spatial distributions for the entire scalp.

A.8 Software implementation and hardware

We performed the ConvNet experiments on Geforce GTX Titan Black GPUs with 6 GB memory. The machines had Intel(R) Xeon(R) E5-2650 v2 CPUs @ 2.60 GHz with 32 cores (which were never fully used as most computations were performed on the GPU) and 128 GB RAM. FBCSP was computed on an Intel(R) Xeon(R) CPU E5-2650 v2 @ 2.60 GHz with 16 cores and 64 GB RAM. We implemented our ConvNets using the Lasagne framework (Dieleman et al., 2015), preprocessing of the data and FBCSP were implemented with the Wyrm library (Venthur et al., 2015). The code used in this study is available under `https://github.com/robintibor/braindecode/`.

References

Antoniades, A., Spyrou, L., Took, C. C., and Sanei, S. (2016). Deep learning for epileptic intracranial EEG data. In *2016 IEEE 26th International Workshop on Machine Learning for Signal Processing (MLSP)*, pages 1–6.

Ball, T., Demandt, E., Mutschler, I., Neitzel, E., Mehring, C., Vogt, K., Aertsen, A., and Schulze-Bonhage, A. (2008). Movement related activity in the high gamma range of the human EEG. *NeuroImage*, 41(2):302–310.

Bashivan, P., Rish, I., Yeasin, M., and Codella, N. (2016). Learning Representations from EEG with Deep Recurrent-Convolutional Neural Networks. In *arXiv:1511.06448 [cs]*. arXiv: 1511.06448.

Blankertz, B., Tomioka, R., Lemm, S., Kawanabe, M., and Muller, K.-R. (2008). Optimizing Spatial filters for Robust EEG Single-Trial Analysis. *IEEE Signal Processing Magazine*, 25(1):41–56.

Brunner, C., Leeb, R., Müller-Putz, G., Schlögl, A., and Pfurtscheller, G. (2008). BCI Competition 2008–Graz data set A. *Institute for Knowledge Discovery (Laboratory of Brain-Computer Interfaces), Graz University of Technology*, pages 136–142.

Buzsáki, G. and Draguhn, A. (2004). Neuronal Oscillations in Cortical Networks. *Science*, 304(5679):1926–1929.

Cecotti, H. and Graser, A. (2011). Convolutional Neural Networks for P300 Detection with Application to Brain-Computer Interfaces. *IEEE Transactions on Pattern Analysis and Machine Intelligence*, 33(3):433–445.

Chin, Z. Y., Ang, K. K., Wang, C., Guan, C., and Zhang, H. (2009). Multi-class filter bank common spatial pattern for four-class motor imagery BCI. In *Annual International Conference of the IEEE Engineering in Medicine and Biology Society, 2009. EMBC 2009*, pages 571–574.

Crone, N. E., Miglioretti, D. L., Gordon, B., and Lesser, R. P. (1998). Functional mapping of human sensorimotor cortex with electrocorticographic spectral analysis. II. Event-related synchronization in the gamma band. *Brain*, 121(12):2301–2315.

Darvas, F., Scherer, R., Ojemann, J. G., Rao, R. P., Miller, K. J., and Sorensen, L. B. (2010). High gamma mapping using EEG. *NeuroImage*, 49(1):930–938.

Dieleman, S., Heilman, M., Kelly, J., Thoma, M., Rasul, D. K., Battenberg, E., Weideman, H., Sønderby, S. K., instagibbs, Britefury, Raffel, C., Degrave, J., peterderivaz, Jon, Fauw, J. D., diogo149, Nouri, D., Schlüter, J., Maturana, D., CongLiu, Olson, E., McFee, B., and takacsg84 (2015). Lasagne: First release. DOI: 10.5281/zenodo.27878.

Galar, M., Fernández, A., Barrenechea, E., Bustince, H., and Herrera, F. (2011). An overview of ensemble methods for binary classifiers in multi-class problems: Experimental study on one-vs-one and one-vs-all schemes. *Pattern Recognition*, 44(8):1761–1776.

Hajinoroozi, M., Mao, Z., Jung, T.-P., Lin, C.-T., and Huang, Y. (2016). EEG-based prediction of driver's cognitive performance by deep convolutional neural network. *Signal Processing: Image Communication*, 47:549–555.

Hammer, J., Pistohl, T., Fischer, J., Kršek, P., Tomášek, M., Marusič, P., Schulze-Bonhage, A., Aertsen, A., and Ball, T. (2016). Predominance of Movement Speed Over Direction in Neuronal Population Signals of Motor Cortex: Intracranial EEG Data and A Simple Explanatory Model. *Cerebral Cortex (New York, NY)*, 26(6):2863–2881.

He, K., Zhang, X., Ren, S., and Sun, J. (2015). Deep Residual Learning for Image Recognition. *arXiv:1512.03385 [cs]*. arXiv: 1512.03385.

Kingma, D. P. and Ba, J. (2014). Adam: A Method for Stochastic Optimization. In *Proceedings of the 3rd International Conference on Learning Representations (ICLR)*.

Lawhern, V. J., Solon, A. J., Waytowich, N. R., Gordon, S. M., Hung, C. P., and Lance, B. J. (2016). EEGNet: A Compact Convolutional Network for EEG-based Brain-Computer Interfaces. *arXiv:1611.08024 [cs, q-bio, stat]*. arXiv: 1611.08024.

Ledoit, O. and Wolf, M. (2004). A well-conditioned estimator for large-dimensional covariance matrices. *Journal of Multivariate Analysis*, 88(2):365–411.

Leeb, R., Brunner, C., Müller-Putz, G., Schlögl, A., and Pfurtscheller, G. (2008). BCI Competition 2008–Graz data set B. *Graz University of Technology, Austria.*

Liang, J., Lu, R., Zhang, C., and Wang, F. (2016). Predicting Seizures from Electroencephalography Recordings: A Knowledge Transfer Strategy. In *2016 IEEE International Conference on Healthcare Informatics (ICHI)*, pages 184–191.

Lotte, F., Congedo, M., Lécuyer, A., Lamarche, F., and Arnaldi, B. (2007). A review of classification algorithms for EEG-based brain–computer interfaces. *Journal of Neural Engineering*, 4(2):R1.

Manor, R. and Geva, A. B. (2015). Convolutional Neural Network for Multi-Category Rapid Serial Visual Presentation BCI. *Frontiers in Computational Neuroscience*, 9:146.

Manor, R., Mishali, L., and Geva, A. B. (2016). Multimodal Neural Network for Rapid Serial Visual Presentation Brain Computer Interface. *Frontiers in Computational Neuroscience*, 10.

Page, A., Shea, C., and Mohsenin, T. (2016). Wearable seizure detection using convolutional neural networks with transfer learning. In *2016 IEEE International Symposium on Circuits and Systems (ISCAS)*, pages 1086–1089.

Quandt, F., Reichert, C., Hinrichs, H., Heinze, H. J., Knight, R. T., and Rieger, J. W. (2012). Single trial discrimination of individual finger movements on one hand: A combined MEG and EEG study. *NeuroImage*, 59(4):3316–3324.

Ren, Y. and Wu, Y. (2014). Convolutional deep belief networks for feature extraction of EEG signal. In *2014 International Joint Conference on Neural Networks (IJCNN)*, pages 2850–2853.

Sakhavi, S., Guan, C., and Yan, S. (2015). Parallel convolutional-linear neural network for motor imagery classification. In *Signal Processing Conference (EUSIPCO), 2015 23rd European*, pages 2736–2740.

Santana, E., Brockmeier, A., and Principe, J. (2014). Joint optimization of algorithmic suites for EEG analysis. In *2014 36th Annual International Conference of the IEEE Engineering in Medicine and Biology Society (EMBC)*, pages 2997–3000.

Schalk, G., McFarland, D. J., Hinterberger, T., Birbaumer, N., and Wolpaw, J. R. (2004). BCI2000: a general-purpose brain-computer interface (BCI) system. *IEEE Transactions on Biomedical Engineering*, 51(6):1034–1043.

Shamwell, J., Lee, H., Kwon, H., Marathe, A. R., Lawhern, V., and Nothwang, W. (2016). Single-trial EEG RSVP classification using convolutional neural networks. In George, T., Dutta, A. K., and Islam, M. S., editors, *SPIE Defense+ Security*, volume 9836. International Society for Optics and Photonics.

Stober, S. (2016). Learning Discriminative Features from Electroencephalography Recordings by Encoding Similarity Constraints. In *Bernstein Conference 2016*.

Stober, S., Cameron, D. J., and Grahn, J. A. (2014). Using Convolutional Neural Networks to Recognize Rhythm Stimuli from Electroencephalography Recordings. In *Proceedings of the 27th International Conference on Neural Information Processing Systems*, NIPS'14, pages 1449–1457, Cambridge, MA, USA. MIT Press.

Sturm, I., Lapuschkin, S., Samek, W., and Müller, K.-R. (2016). Interpretable deep neural networks for single-trial EEG classification. *Journal of Neuroscience Methods*, 274:141–145.

Sun, G., Hu, J., and Wu, G. (2010). A novel frequency band selection method for Common Spatial Pattern in Motor Imagery based Brain Computer Interface.

Sun, X., Qian, C., Chen, Z., Wu, Z., Luo, B., and Pan, G. (2016). Remembered or Forgotten?—An EEG-Based Computational Prediction Approach. *PLOS ONE*, 11(12):e0167497.

Tabar, Y. R. and Halici, U. (2017). A novel deep learning approach for classification of EEG motor imagery signals. *Journal of Neural Engineering*, 14(1):016003.

Tang, Z., Li, C., and Sun, S. (2017). Single-trial EEG classification of motor imagery using deep convolutional neural networks. *Optik - International Journal for Light and Electron Optics*, 130:11–18.

Thodoroff, P., Pineau, J., and Lim, A. (2016). Learning Robust Features using Deep Learning for Automatic Seizure Detection. In *JMLR Workshop and Conference Proceedings*, volume 56.

Venthur, B., Dähne, S., Höhne, J., Heller, H., and Blankertz, B. (2015). Wyrm: A Brain-Computer Interface Toolbox in Python. *Neuroinformatics*, pages 1–16.

Wang, Z., Lyu, S., Schalk, G., and Ji, Q. (2013). Deep Feature Learning Using Target Priors with Applications in ECoG Signal Decoding for BCI. In *Proceedings of the Twenty-Third International Joint Conference on Artificial Intelligence*, IJCAI '13, pages 1785–1791, Beijing, China. AAAI Press.
